# Coupling photodynamic hypoxia amplification with a self-reporting chemiluminescent prodrug for adaptive precision therapy

**DOI:** 10.1016/j.mtbio.2026.103712

**Published:** 2026-09-24

**Authors:** Jie Gao, Lianzhu Wei, Yujie Wang, Maocheng Yang, Yumei Wu, Wen-Chao Geng, Zeli Yuan

**Affiliations:** a School of Pharmacy, Key Laboratory of Basic Pharmacology of Ministry of Education and Joint International Research Laboratory of Ethnomedicine of Ministry of Education, Guizhou International Science & Technology Cooperation Base of Medical Optical Theranostics Research, New Drug Discovery and Evaluation Center for International Cooperation and Disciplinary Innovation (“111 Center”), Zunyi Medical University, Zunyi, Guizhou, 563003, China; b College of Life Science and Technology, Beijing University of Chemical Technology, Beijing, 100029, China

**Keywords:** Chemiluminescence, Photodynamic therapy, Hypoxia-activated prodrugs, Self-reporting, Azoreductase

## Abstract

Hypoxia is a hallmark of solid tumors that provides a therapeutic window for hypoxia-activated prodrugs (HAPs). However, the therapeutic performance of HAPs is often limited by heterogeneous tumor hypoxia and the lack of real-time feedback on prodrug activation. Herein, we report a self-reporting nanomedicine, CL-Azo-CPT/Ce6 NPs, that integrates hypoxia amplification with chemiluminescent monitoring in a “Sense-Amplify-Report” strategy. The system combines a photosensitizer, chlorin e6 (Ce6), with a hypoxia-responsive chemiluminescent prodrug, CL-Azo-CPT, in which camptothecin (CPT) is linked to a Schaap's dioxetane scaffold through an azobenzene (azo) trigger. Upon irradiation, Ce6-mediated photodynamic therapy consumes local oxygen and aggravates tumor hypoxia, thereby promoting azoreductase-mediated azo cleavage and accelerating CPT release. This activation process is accompanied by chemiluminescence emission, the intensity of which shows a stoichiometric correlation with drug release and thus provides an excitation-free, real-time readout of prodrug activation. *In vitro* studies and evaluation in a murine peritoneal metastasis model demonstrated enhanced antitumor efficacy, marked tumor regression, and prolonged survival. This work establishes a hypoxia-amplified and self-reporting chemo-photodynamic platform that addresses two central limitations of HAPs, namely insufficient activation and the absence of in vivo activation feedback.

## Highlights

• A self-reporting chemiluminescent prodrug nanoplatform was developed.
• PDT-driven oxygen depletion amplified tumor hypoxia and prodrug activation.
• Chemiluminescence stoichiometrically reported CPT release in real time.
• Excitation-free imaging visualized in vivo prodrug activation in tumors.
• Closed-loop chemo-photodynamic therapy improved tumor regression and survival.

## Graphical abstract

**Figure:**
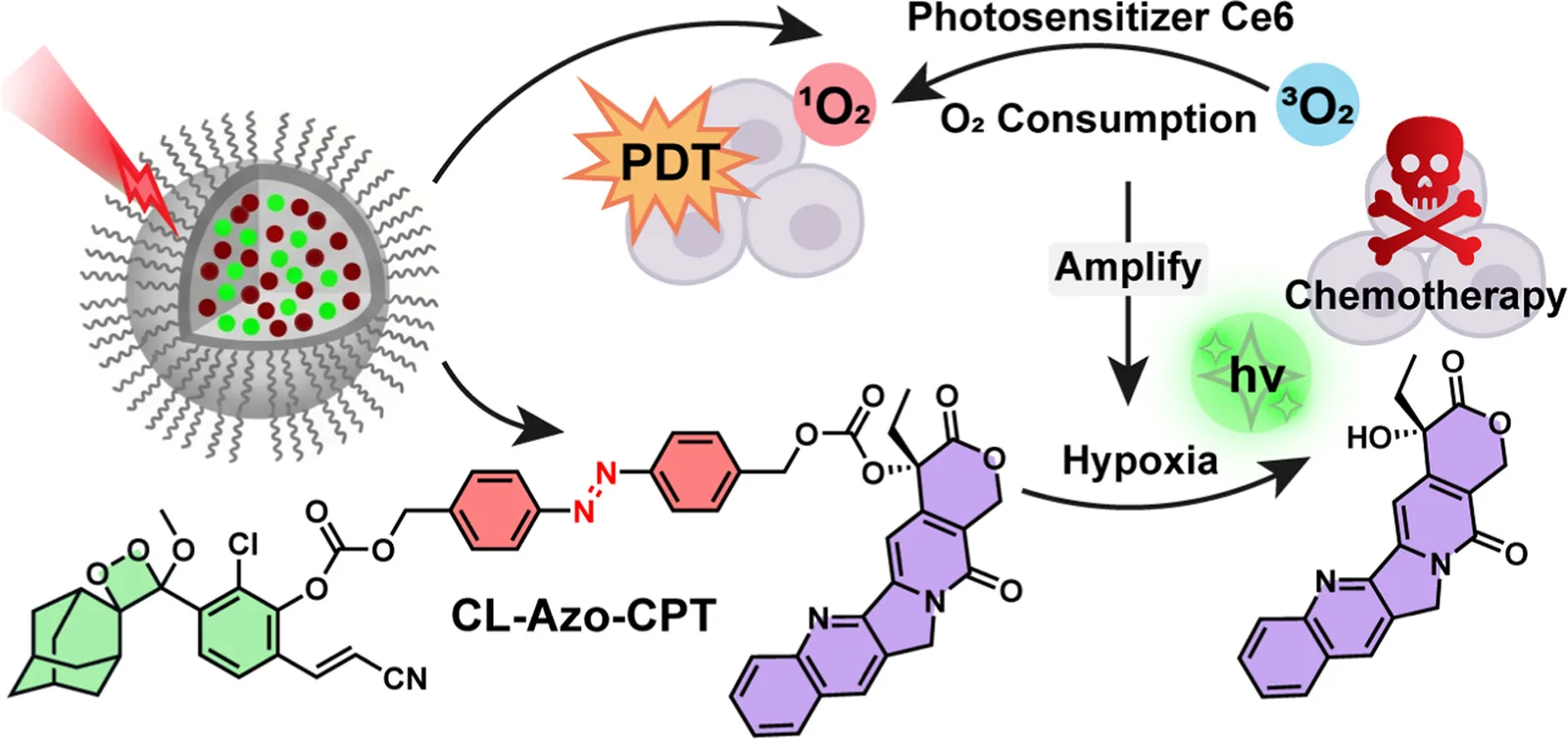

## 1 Introduction

Hypoxia is a defining feature of most solid tumors, arising from the imbalance between oxygen supply and consumption in rapidly proliferating tissues [[1], [2], [3], [4], [5]]. This pathological microenvironment has long motivated the development of hypoxia-activated prodrugs (HAPs), which exploit reductive enzymes upregulated under hypoxia to selectively release cytotoxic agents at tumor sites [[6], [7], [8], [9], [10], [11], [12], [13], [14], [15]]. In practice, however, the therapeutic performance of HAPs is often limited by the spatial and temporal heterogeneity of tumor hypoxia [16]. The clinical failure of representative candidates such as evofosfamide illustrates this challenge: only a fraction of tumor cells experiences hypoxia severe enough to trigger efficient prodrug activation, whereas less hypoxic regions remain insufficiently treated and continue to drive disease progression. Overcoming this limitation requires HAP systems that can not only respond to endogenous hypoxia, but also amplify it and report the extent of drug activation in real time.

Chemiluminescence (CL) offers an attractive solution to the long-standing problem of activation monitoring [[17], [18], [19], [20], [21]]. Because CL does not require external excitation, it avoids tissue autofluorescence and minimizes background interference, thereby providing high signal-to-noise readouts in living tissues [[22], [23], [24], [25], [26], [27], [28], [29]]. A seminal study by Shabat and co-workers established that Schaap's dioxetane can serve as a chemiluminescent prodrug scaffold, allowing a single activation event to produce both drug release and photon emission [30]. Building on this concept, we previously developed CPT-NBz-CL, a nitroreductase-responsive CL prodrug that couples camptothecin (CPT) release with luminescent output in a murine peritoneal carcinomatosis model [31]. More recently, Yang and co-workers extended this strategy to azoreductase (AZR) by constructing an azobenzene-linked CL prodrug for breast cancer imaging and therapy [32]. These studies demonstrate that CL can function as a faithful reporter of prodrug activation. However, existing CL theranostic systems remain essentially passive: they can visualize activation only after hypoxia has already occurred, but cannot modulate the tumor microenvironment to improve activation efficiency.

Photodynamic therapy (PDT) provides a complementary means to address this limitation [[33], [34], [35], [36]]. Upon light irradiation, photosensitizers such as chlorin e6 (Ce6) generate reactive oxygen species while simultaneously consuming molecular oxygen, thereby inducing oxidative damage and aggravating local hypoxia. This property has been exploited to enhance the activation of hypoxia-responsive therapeutics by co-delivering photosensitizers and prodrugs in nanocarriers, leading to improved chemo-photodynamic efficacy [[37], [38], [39], [40], [41], [42], [43], [44], [45], [46], [47]]. Although PDT can intensify hypoxia and promote prodrug activation, the extent and kinetics of this process usually remain inaccessible in vivo. As a result, treatment is still administered in an open-loop manner, without direct feedback on whether hypoxia amplification has translated into efficient drug release.

Herein, we introduce CL-Azo-CPT/Ce6 NPs, a self-reporting chemiluminescent nanoplatform that integrates photodynamic hypoxia amplification with real-time luminescent feedback via a “Sense-Amplify-Report” cycle (Scheme 1). The molecular design of chemiluminescent prodrug CL-Azo-CPT features a dioxetane luminophore linked to CPT through a self-immolative carbonate spacer and an AZR-responsive azobenzene (azo) trigger. Co-encapsulation with Ce6 in DSPE-mPEG2000 nanoparticles enables a cascade process in which basal tumor hypoxia initiates azo cleavage, leading to CPT release and concomitant CL emission (Sense), whereas light-activated Ce6 further consumes oxygen and aggravates hypoxia (Amplify), thereby promoting AZR-mediated prodrug activation. This process produces additional CPT together with CL photons whose intensity shows a stoichiometric correlation with drug release (Report). The resulting CL signal therefore provides an excitation-free, real-time readout of prodrug activation and offers a basis for feedback-guided optimization of subsequent light dosing. Using CT26 and HCT116 cells and a CT26-Luc1 peritoneal metastasis model, we demonstrate that this integrated strategy significantly improves antitumor efficacy and prolongs survival, establishing a framework for hypoxia-amplified and self-reporting chemo-photodynamic therapy. The central advance of this work is therefore the integration of three functions within a single formulation, namely photodynamic hypoxia amplification, hypoxia-triggered prodrug activation, and self-reporting of the activation event, rather than the combination of photodynamic and chemotherapeutic modalities alone.

**Scheme 1:**
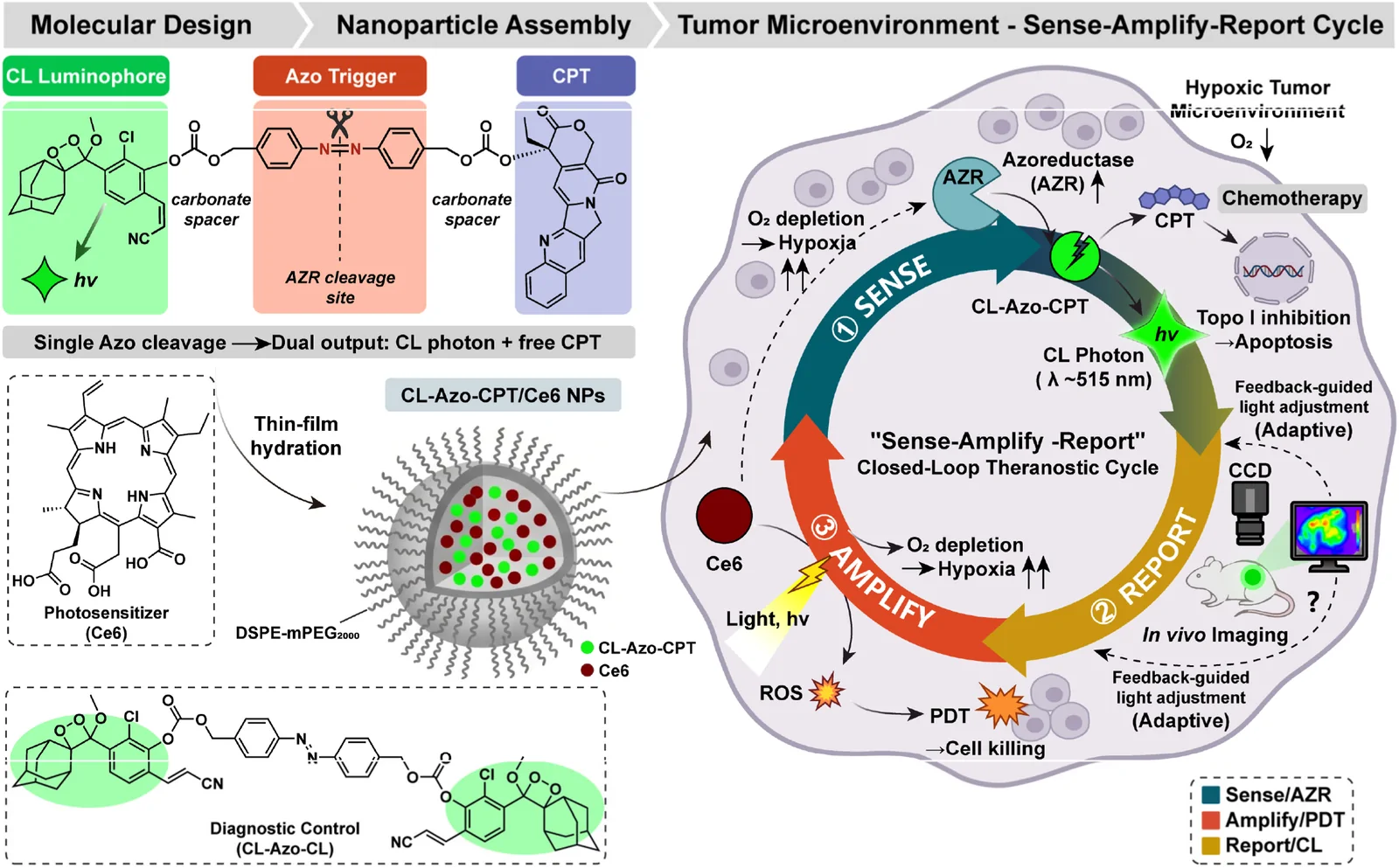
Schematic illustration of the “Sense-Amplify-Report” self-reporting mechanism of CL-Azo-CPT/Ce6 NPs. The chemiluminescence readout reports prodrug activation in real time and provides a basis for feedback-guided decisions on subsequent light dosing; the current formulation does not perform automated real-time dose adjustment.

## 2 Results and discussion

### 2.1 Molecular design and self-reporting activation of CL-azo-CPT

The self-reporting prodrug CL-Azo-CPT was designed to couple hypoxia-responsive activation with chemiluminescent signal generation within a single covalent framework (Synthetic route as shown in Fig. 1A). Schaap's adamantylidene-1,2-dioxetane luminophore was linked to the anticancer agent camptothecin (CPT) through an azoreductase (AZR)-responsive azobenzene trigger and a self-immolative carbonate spacer. Upon bioreductive cleavage of the azo bond, the molecule undergoes a self-immolative cascade that simultaneously releases CPT and activates the chemiluminescent luminophore. This design establishes a direct stoichiometric correlation between photon emission and drug release, such that the luminescence signal serves as a molecular-level readout of prodrug activation. For comparison of the optical behavior, a symmetric diagnostic control, CL-Azo-CL, bearing two dioxetane units flanking the same azo core, was synthesized in parallel (Scheme S1). Both compounds were fully characterized by ^1^H NMR, ^13^C NMR, and high-resolution mass spectrometry (Fig. S1–S15). High-performance liquid chromatography (HPLC) analysis further verified that the purity of the CL-Azo-CPT prodrug was >98% (Fig. S16).

**Fig. 1:**
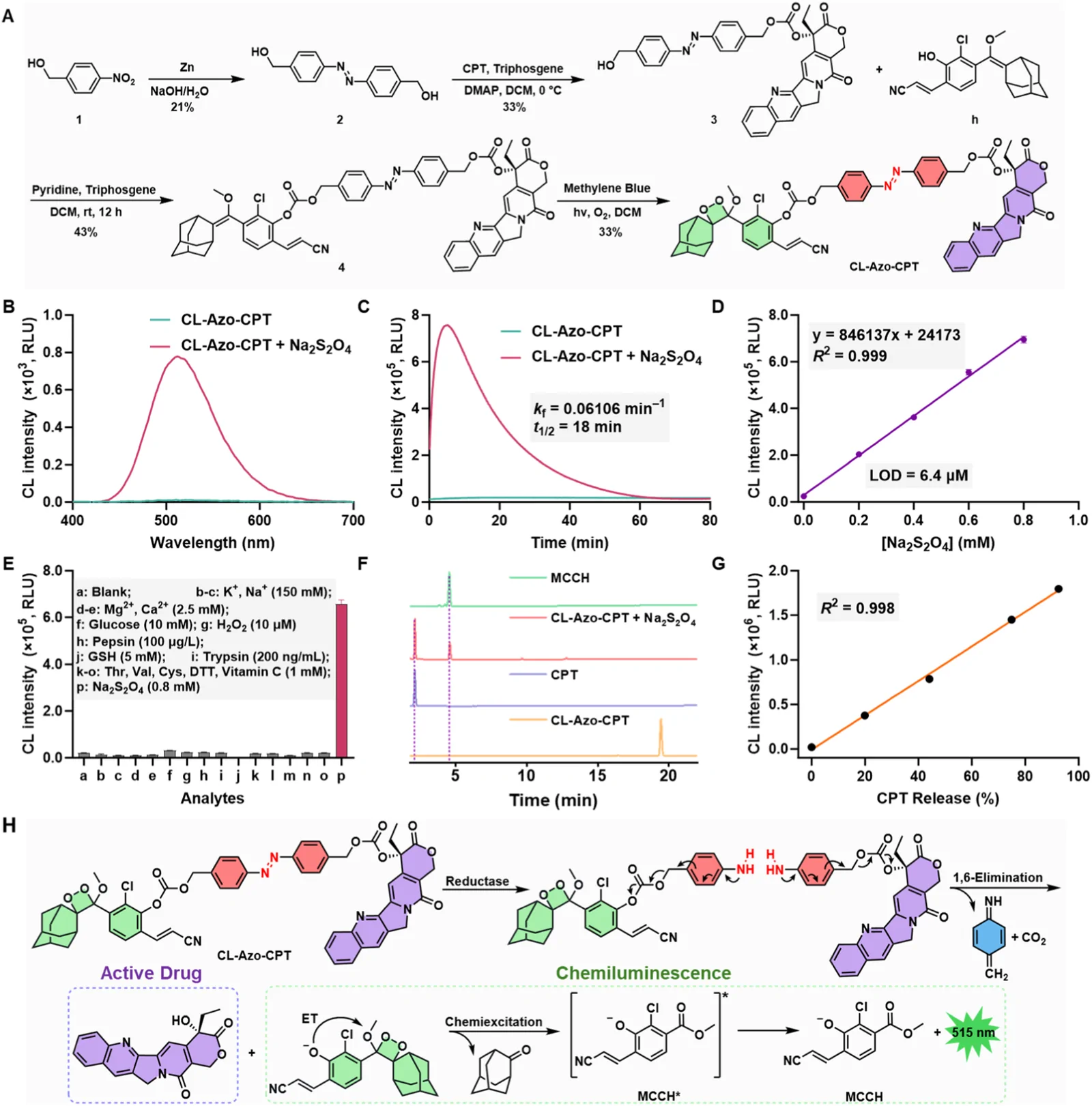
Design, synthesis, and chemiluminescent validation of the self-reporting prodrug CL-Azo-CPT. (A) Synthetic route to CL-Azo-CPT. (B) CL spectra of CL-Azo-CPT (10 μM) with and without Na_2_S_2_O_4_ (5 mM). (C) CL kinetic profiles of CL-Azo-CPT (10 μM) in Tris-HCl buffer (pH 7.4) with and without Na_2_S_2_O_4_ (5 mM). (D) Linear correlation between the CL intensity and Na_2_S_2_O_4_ concentration (mean ± SD, *n* = 3). (E) Selectivity of the CL response of CL-Azo-CPT (10 μM) toward various biologically relevant analytes and enzymes (mean ± SD, *n* = 3). (F) HPLC chromatograms monitoring the Na_2_S_2_O_4_-triggered release of CPT. (G) Correlation plot between CPT release quantified by HPLC and the corresponding CL intensity. (H) Proposed hypoxia-triggered activation and self-reporting mechanism of CL-Azo-CPT.

The reporting function of this design relies on chemiluminescence generated upon prodrug activation. We first evaluated the CL emission spectra to confirm the signal unmasking. In the presence of sodium dithionite (Na_2_S_2_O_4_), used here as a chemical mimic of AZR activity, CL-Azo-CPT exhibited a broad emission spanning 430–650 nm with a maximum at 515 nm, corresponding to an approximately 90-fold enhancement relative to the background in the absence of reductant (Fig. 1B). CL-Azo-CL showed a similar spectral profile and a comparable signal enhancement (Fig. S17A). No appreciable chemiluminescence was detected without Na_2_S_2_O_4_, indicating that the intact azo moiety efficiently suppresses dioxetane emission prior to activation. We next examined the temporal evolution of the CL signal to assess its suitability for dynamic monitoring. Upon addition of Na_2_S_2_O_4_, the signal from CL-Azo-CPT rapidly increased to a maximum within 5 min, representing an approximately 35-fold enhancement over the untreated control, and then decayed with a rate constant of 0.061 min^−1^ and a half-life (*t*_1/2_) of 18 min (Fig. 1C). The control compound CL-Azo-CL exhibited faster kinetics, reaching maximum intensity within 3 min with a 70-fold signal increase, a decay rate constant of 0.089 min^−1^, and a half-life of 15 min (Fig. S17B). Furthermore, concentration-dependent measurements showed that the chemiluminescence intensity of CL-Azo-CPT increased to a maximum and then decayed with increasing activation time, consistent with the kinetic behavior described above (Fig. S18).

A linear correlation between CL intensity and Na_2_S_2_O_4_ concentration was observed over the 0–0.8 mM range for both CL-Azo-CPT and CL-Azo-CL (*R*^2^ = 0.999), with limits of detection (LOD) of 6.4 μM and 3.1 μM, respectively (Fig. 1D and S19A). Selectivity assays against a panel of biologically relevant species, including metal ions (Na^+^, K^+^, Mg^2+^, Ca^2+^), amino acids (glutathione (GSH), cysteine (Cys), threonine (Thr), valine (Val)), reactive oxygen species (H_2_O_2_), reducing agents (dithiothreitol (DTT), vitamin C), glucose, and enzymes (pepsin, trypsin), showed that only the azo-reductive trigger elicited a pronounced CL response (Fig. 1E and S19B). These results indicate that the reporting signal is highly specific and is unlikely to be substantially perturbed by other reductive or reactive species present in the tumor microenvironment.

The stoichiometric relationship between chemiluminescent reporting and drug release was further established by HPLC analysis. As Na_2_S_2_O_4_ concentration increased, the peak corresponding to CL-Azo-CPT (*t*_R_ = 19.45 min) progressively decreased, while two new peaks assigned to CPT (*t*_R_ = 2.13 min) and MCCH (the chemiluminescent product, *t*_R_ = 4.60 min) emerged and increased in parallel (Fig. 1F and S20). The concerted formation of CPT and MCCH, together with the consumption of the parent prodrug, confirms that activation of CL-Azo-CPT simultaneously generates the therapeutic payload and the reporting species. Notably, the CL intensity showed an excellent linear correlation with the extent of CPT release quantified by HPLC (*R*^2^ = 0.997, Fig. 1G), providing direct evidence that photon output quantitatively reflects prodrug activation. On this basis, the activation of CL-Azo-CPT can be described as a three-step process: bioreduction of the azo bond, spontaneous self-immolative fragmentation, and concomitant release of CPT with chemiluminescence emission (Fig. 1H).

As supplementary evidence, the fluorescence properties of CL-Azo-CPT and CL-Azo-CL were also examined. In the absence of Na_2_S_2_O_4_, both CL-Azo-CPT and CL-Azo-CL exhibited negligible fluorescence, indicating that the intact azo moiety effectively quenches the luminophore's secondary fluorescent reporting channel. Upon addition of Na_2_S_2_O_4_, fluorescence derived from MCCH was dramatically recovered, confirming that the same reductive cleavage event that triggers CL emission also restores fluorescence (Fig. S21).

### 2.2 Nanoparticle assembly and photodynamic activity of CL-azo-CPT/Ce6 NPs

To construct the hypoxia-regulable formulation, CL-Azo-CPT was co-encapsulated with the photosensitizer Ce6 into DSPE-mPEG_2000_ nanoparticles via thin-film hydration. Using ultrafiltration and HPLC quantification, the encapsulation efficiency and drug-loading capacity of the CL-Azo-CPT/Ce6 NPs were determined to be 97.33 ± 2.08% and 3.65 ± 0.08% for CL-Azo-CPT, and 65.33 ± 4.16% and 0.92 ± 0.17% for Ce6, respectively. Dynamic light scattering (DLS) analysis showed that CL-Azo-CPT/Ce6 NPs had an average hydrodynamic diameter of 169 nm with a polydispersity index (PDI) of 0.22 (Fig. 2A). Transmission electron microscopy (TEM) further confirmed a uniform spherical morphology (Fig. 2B). The nanoparticles remained stable over 7 days at 4 °C, with only minimal variation in particle size and PDI values consistently below 0.3 (Fig. 2C), indicating adequate colloidal stability for subsequent biological studies. Control formulations, including CL-Azo-CPT NPs (158 nm, PDI = 0.25), Ce6 NPs (265 nm, PDI = 0.29), and CL-Azo-CL NPs (163 nm, PDI = 0.23), were prepared in parallel for mechanistic deconvolution (Fig. S22 and S23). The absorption and fluorescence spectra recorded on the CL-Azo-CPT/Ce6 NPs themselves show maxima at 654 nm and 662 nm, respectively, matching those of free Ce6 (Fig. S24), which confirms that the fluorescence-imaging capability of Ce6 is preserved after co-encapsulation and defines the imaging parameters used in the cellular studies.

**Fig. 2:**
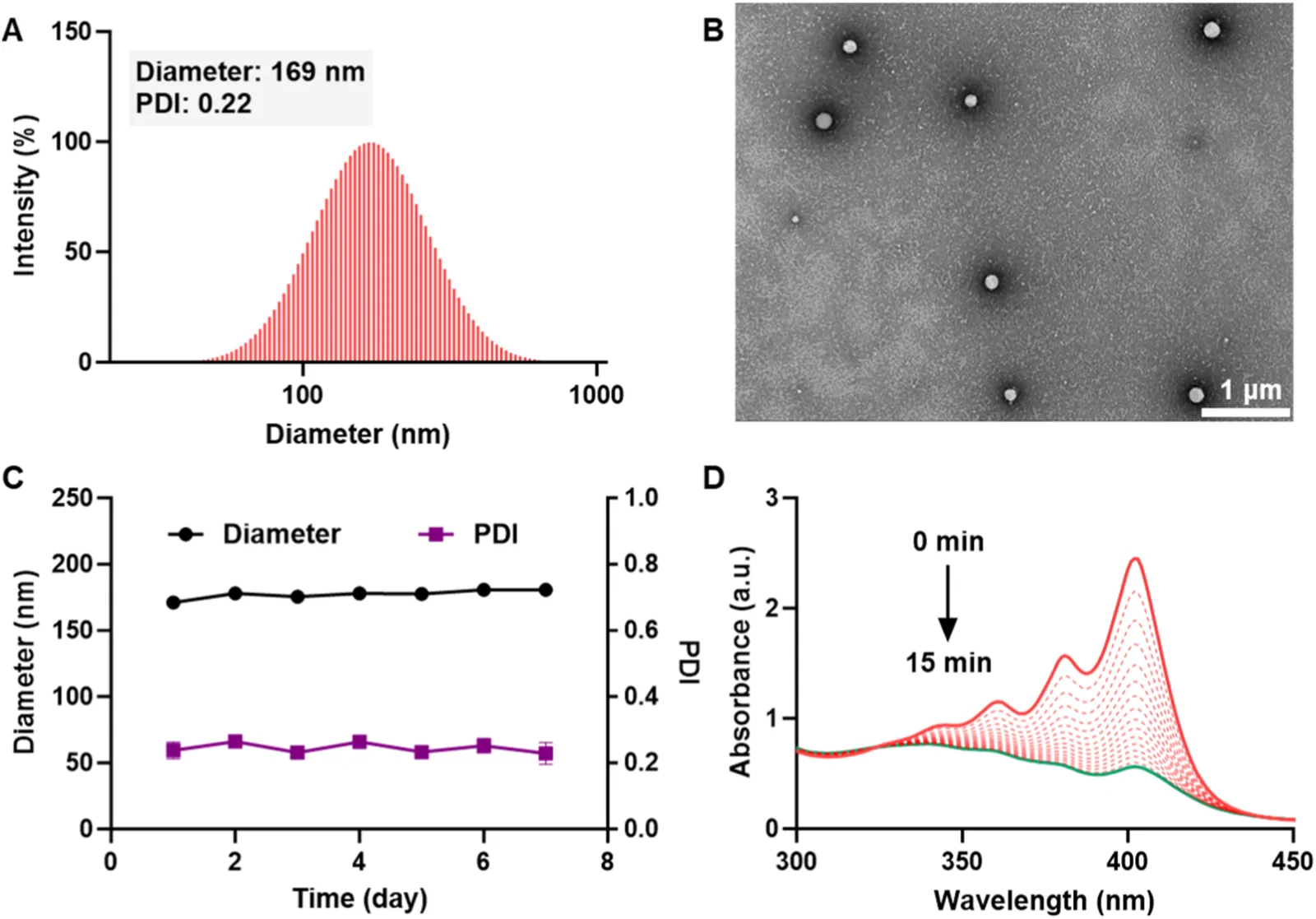
Characterization and singlet oxygen generation capability of CL-Azo-CPT/Ce6 NPs. (A) Hydrodynamic size distribution of CL-Azo-CPT/Ce6 NPs determined by DLS. (B) Representative TEM image of CL-Azo-CPT/Ce6 NPs (scale bar = 1 μm). (C) Colloidal stability of CL-Azo-CPT/Ce6 NPs stored in Tris-HCl buffer at 4 °C over 7 days (mean ± SD, *n* = 3). (D) Time-dependent absorption spectra monitoring the decay of ABDA (50 μM) in the presence of CL-Azo-CPT/Ce6 NPs (10 μM/10 μM) with ABDA (50 μM) upon white light irradiation (0.8 W/cm^2^) for 15 min.

We next examined whether nanoencapsulated Ce6 retained its photodynamic activity using 9,10-anthracenediylbis(methylene)dimalonic acid (ABDA) as a singlet oxygen indicator. Under white-light irradiation (0.8 W/cm^2^, 15 min), the ABDA absorbance at 402 nm decreased rapidly in the presence of free Ce6, Ce6 NPs, and CL-Azo-CPT/Ce6 NPs, whereas no appreciable change was observed for ABDA alone (Fig. 2D and S25). These results indicate that the PEGylated nanoformulation preserves the ability of Ce6 to generate singlet oxygen under irradiation, and that co-encapsulation of CL-Azo-CPT does not measurably impair the photodynamic performance of the photosensitizer. Given that photodynamic action is accompanied by oxygen consumption, this retained activity provides the photochemical basis for the hypoxia-amplifying role proposed for CL-Azo-CPT/Ce6 NPs, which was further evaluated in the subsequent biological studies.

### 2.3 Cellular validation of the Sense-Amplify-Report cascade

Having established the molecular and photochemical properties of the system, we next examined whether the Sense-Amplify-Report cascade operates coherently in living cells. Confocal laser scanning microscopy (CLSM) of CT26 and HCT116 cells treated with CL-Azo-CPT/Ce6 NPs under CoCl_2_-induced hypoxia (CT26: 0.2 mM; HCT116: 1 mM; both conditions maintained cell viability above 80%; Fig. S26) showed a time-dependent increase in the intracellular fluorescence signals assigned to CPT and the MCCH reporter over 0–6 h, with maximal intensities observed at 6 h (Fig. S27 and S28). By contrast, both signals remained weak under normoxic conditions. The fluorescence of Ce6 was largely unchanged irrespective of oxygen status. These results indicate that hypoxia promotes intracellular prodrug activation, leading to the concurrent generation of CPT and the reporter species. Consistently, CL imaging showed clear time-dependent (0–6 h) and concentration-dependent (10–50 μM) signal increases in hypoxic cells (Fig. 3A and S29–S30), further supporting the use of CL output as a readout of intracellular activation.

**Fig. 3:**
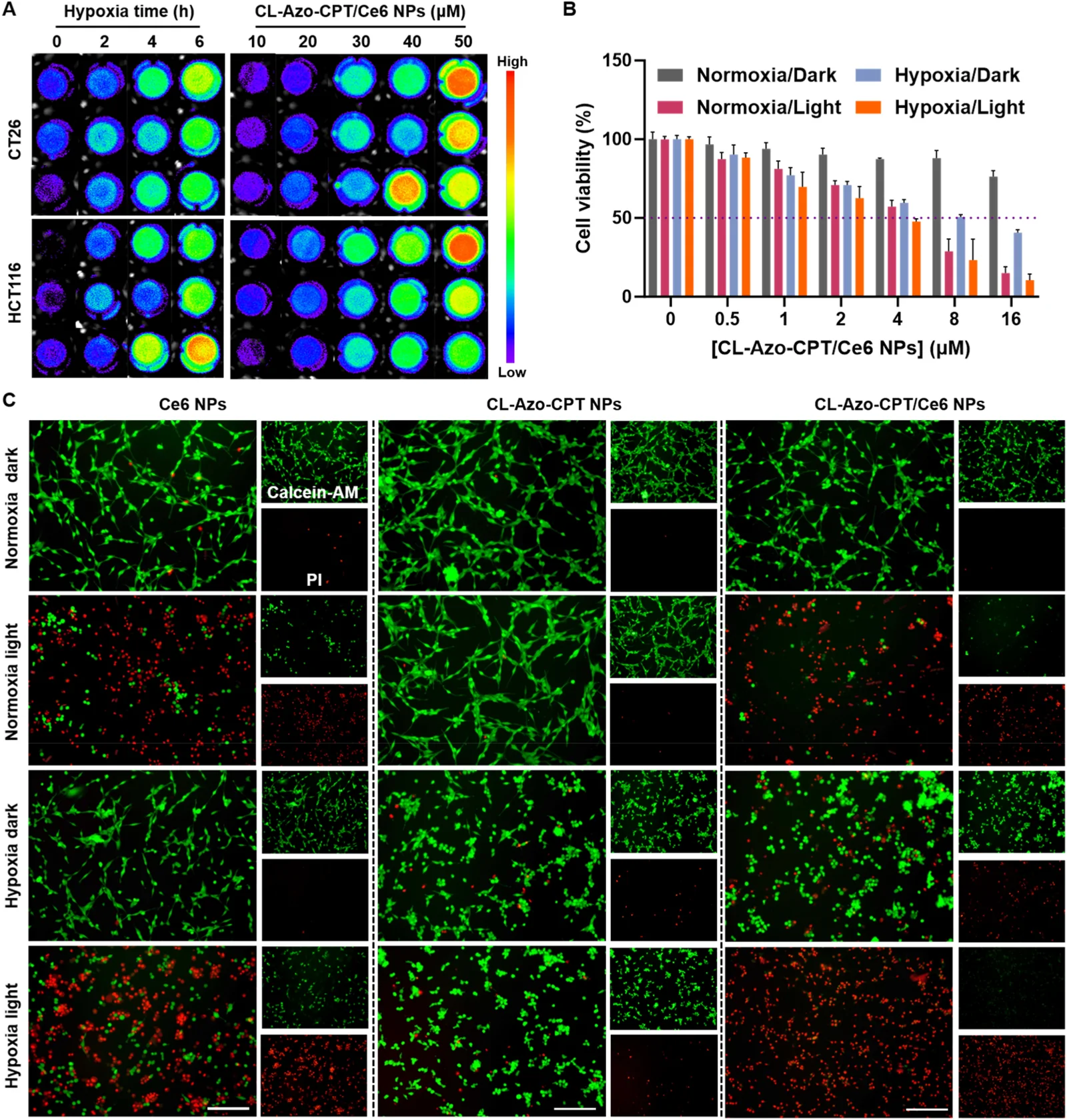
*In vitro* CL imaging and cytotoxicity of CL-Azo-CPT/Ce6 NPs. (A) CL images of CT26 and HCT116 cells treated with CL-Azo-CPT/Ce6 NPs (20 μM) over different hypoxia durations (0–6 h), and cells treated with varying concentrations (10–50 μM) under hypoxic conditions. Exposure time: 60 s (*n* = 3). (B) Cell viability of CT26 cells treated with CL-Azo-CPT/Ce6 NPs under different conditions with light irradiation (220.7 mW/cm^2^, 30 s) or in the dark (mean ± SD, *n* = 6). (C) Representative fluorescence images of CT26 cells after different treatments under light irradiation (220.7 mW/cm^2^, 30 s) or in the dark, stained with Calcein-AM (green, live cells) and PI (red, dead cells). Scale bar: 200 μm.

We next assessed whether Ce6 retained photodynamic activity under different oxygen and irradiation conditions. Intracellular ROS generation was monitored using 2′,7′-dichlorodihydrofluorescein diacetate (DCFH-DA) under four conditions defined by oxygen status and light exposure (normoxia/hypoxia × dark/light; Fig. S31 and S32). Under normoxia with light irradiation, both Ce6 NPs and CL-Azo-CPT/Ce6 NPs produced strong DCF fluorescence, confirming efficient ROS generation. As expected, negligible ROS signals were observed in the dark under either oxygen condition. Under hypoxia with light irradiation, ROS generation by CL-Azo-CPT/Ce6 NPs remained detectable, although reduced relative to that under normoxia. These results indicate that Ce6 preserves measurable photodynamic activity even under oxygen-limited conditions, thus providing the basis for the light-triggered enhancement step in the cascade.

To delineate the therapeutic contributions of the individual components, MTT assays were performed in CT26 and HCT116 cells under the same four-condition matrix (normoxia/hypoxia × dark/light). Free CPT exhibited intrinsic, environment-independent cytotoxicity (Fig. S33, IC_50_ in CT26/HCT116: 4.7/5.9 μM under normoxia; 7.9/3.2 μM under hypoxia). The drug-free reporter CL-Azo-CL NPs maintained cell viabilities above 80% in all groups (Fig. S33), indicating that the chemiluminescent scaffold itself is essentially non-cytotoxic. Under normoxic dark conditions, CL-Azo-CPT NPs, Ce6 NPs, and CL-Azo-CPT/Ce6 NPs all showed minimal toxicity (IC50 ≫ 16 μM; Fig. 3B and S35–S37; Table S1), confirming that CPT remained largely caged and that Ce6 was inactive in the absence of irradiation.

The individual “Sense” and “Amplify” elements became evident only under their corresponding activating conditions. Under normoxic light conditions, Ce6 NPs and CL-Azo-CPT/Ce6 NPs exhibited potent PDT-mediated cell death (IC_50_ = 6.2 and 4.2 μM in CT26; 4.4 and 3.8 μM in HCT116), whereas CL-Azo-CPT NPs remained largely inactive (IC_50_ ≫ 16 μM), confirming PDT specificity. Conversely, under hypoxic dark conditions (the “Sense” module alone), both CL-Azo-CPT NPs and CL-Azo-CPT/Ce6 NPs showed moderate cytotoxicity (IC50 = 17.5 and 8.2 μM in CT26; 14.6 and 6.8 μM in HCT116), validating the successful hypoxia-triggered azo cleavage and subsequent CPT liberation. As expected, PDT alone was severely compromised by oxygen deprivation, with the IC50 of Ce6 NPs increasing 2-fold under hypoxia with light irradiation (8.8 μM in CT26; 7.3 μM in HCT116).

Under the dual-trigger condition of hypoxia and light irradiation, CL-Azo-CPT/Ce6 NPs showed the highest potency among all treatments, affording the lowest IC50 values in both cell lines (3.0 μM in CT26; 2.6 μM in HCT116; Fig. 3B and S35–S37). This result indicates that coupling hypoxia-responsive chemotherapy with photodynamic activation produces a substantially stronger antitumor effect than either modality alone. Live/dead staining further supported this conclusion, as near-complete cell ablation was observed only for CL-Azo-CPT/Ce6 NPs under hypoxia with light irradiation (Fig. 3C and S38). Notably, the same activation process that enhanced cytotoxicity also generated concentration- and time-dependent chemiluminescence signals, thereby linking therapeutic activation with optical reporting. Together, these cellular results support the operation of a coordinated “Sense-Amplify-Report” cascade, in which hypoxia initiates prodrug activation and CL emission, while light-triggered Ce6 activation further strengthens the therapeutic response.

### 2.4 *In vivo* chemiluminescent imaging of prodrug activation

To examine whether light treatment enhances prodrug activation in vivo, CL-Azo-CPT/Ce6 NPs were locally injected into subcutaneous 4T1 tumors and contralateral normal tissues, followed by either white-light irradiation for 10 min or dark treatment. In tumors without irradiation, endogenous hypoxia induced a rapid CL response that reached a maximum at 50 min and then gradually declined (Fig. 4A and B). Upon light irradiation, the CL profile shifted to a later but stronger maximum at 110 min, together with a more sustained emission window (Fig. 4A and B). The peak CL intensity and integrated signal in irradiated tumors were 1.69- and 1.86-fold higher, respectively, than those in the non-irradiated group (Fig. 4C). By contrast, normal tissues showed consistently weak CL signals and no comparable enhancement upon irradiation (Fig. 4C). Given the stoichiometric correlation between CL output and CPT release established *in vitro*, these results support that photodynamic treatment promotes prodrug activation in tumors and that this process can be captured by CL imaging in vivo. It should be noted that the strict stoichiometric correlation between CL intensity and CPT release was established at the molecular level under well-defined solution conditions. In living tissues, absorption and scattering attenuate the emitted photons, so the in vivo CL intensity should be interpreted as a relative, semi-quantitative indicator of the extent of prodrug activation rather than an absolute measure of the released drug amount.

**Fig. 4:**
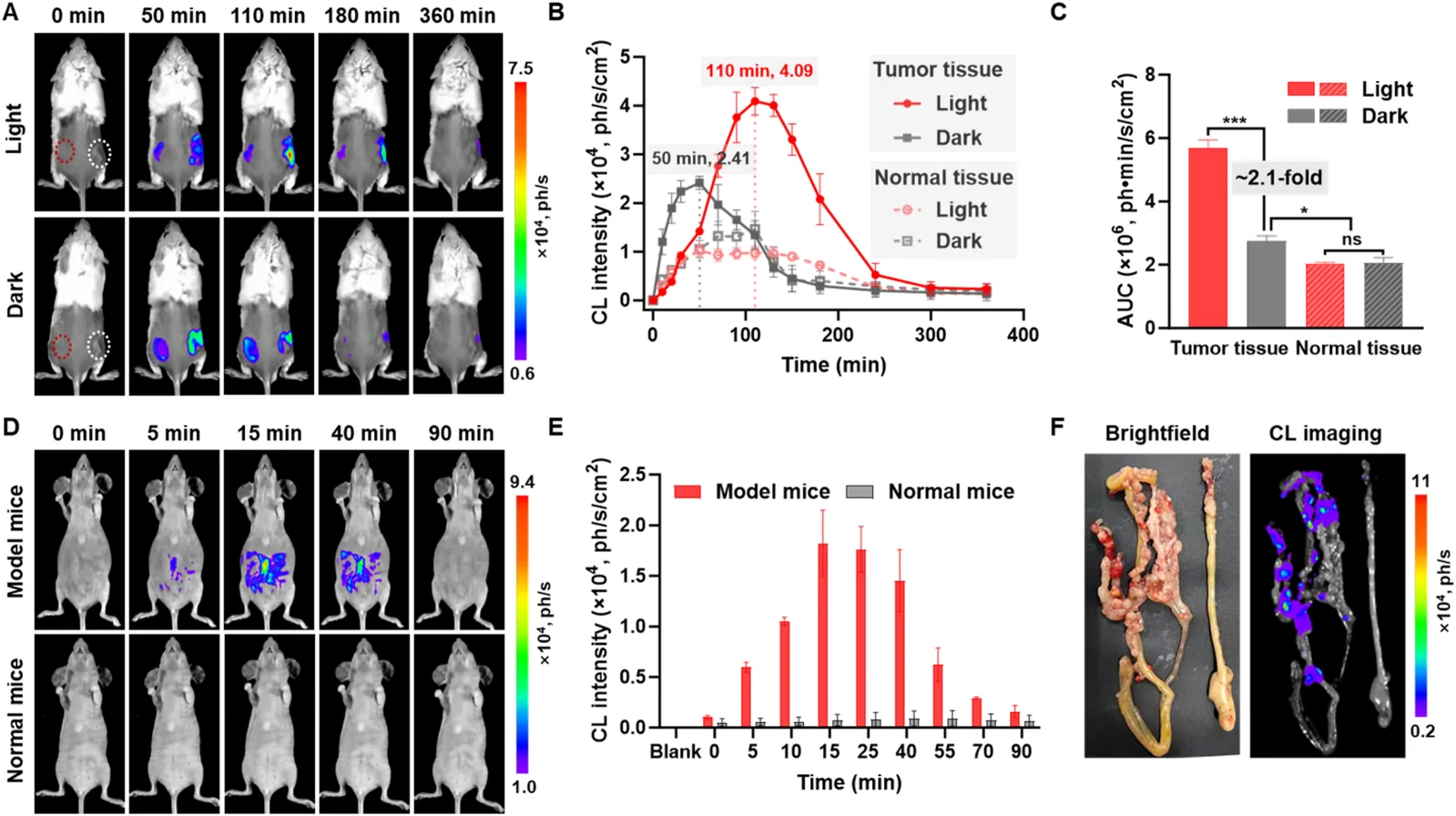
*In vivo* validation of the PDT-amplified “Amplify” and tumor-specific “Report” functions of CL-Azo-CPT/Ce6 NPs. (A) *In vivo* CL images of tumors (white circles) and normal tissues (red circles) at indicated time points after *in situ* injection of CL-Azo-CPT/Ce6 NPs (100 μM, 50 μL) under light irradiation (0.8 W/cm^2^, 10 min) or dark conditions. (B) Corresponding time-dependent CL signal intensities and (C) area under the curve (AUC) analyses (mean ± SD, *n* = 3). (D) *In vivo* CL imaging of peritoneal tumor-bearing mice and healthy control mice at indicated time points following intraperitoneal injection of CL-Azo-CPT/Ce6 NPs (100 μM, 200 μL). (E) Quantitative CL intensities from the corresponding abdominal regions over time (mean ± SD, *n* = 3). (F) *Ex vivo* bright-field and CL images of the excised intestines at 15 min post-injection. (CL acquisition exposure time: 60 s).

We next assessed the self-reporting behavior of the system in a CT26-Luc1 peritoneal metastasis model, in which diffuse intraperitoneal lesions provide a relevant setting for non-invasive monitoring of drug activation. Following intraperitoneal administration of CL-Azo-CPT/Ce6 NPs (100 μM, 200 μL), tumor-bearing mice showed a distinct CL signal that peaked at 15 min and gradually decreased over 90 min (Fig. 4D and E; representative images at all time points are shown in Fig. S39). Under the same conditions, no detectable CL signal was observed in healthy mice, indicating that CL emission was associated with the tumor microenvironment rather than nonspecific decomposition (Fig. 4D and E and Fig. S39). CL-Azo-CPT NPs (without Ce6) produced a similar CL time course, with a peak at 25 min, indicating that co-encapsulation of Ce6 did not compromise the reporting function of the prodrug (Fig. S40 and S41). The spatial distribution of the CL signal was broadly consistent with the bioluminescence pattern of luciferase-expressing CT26-Luc1 tumors imaged with diphenylterazine (DTZ) (Fig. S42 and S43), although the CL signal reflects regional prodrug activation rather than direct tumor cell labeling. *Ex vivo* imaging at 15 min after administration further confirmed that CL emission was localized to intestinal tumor nodules (Fig. 4F). Taken together, these results show that CL-Azo-CPT/Ce6 NPs enable excitation-free imaging of prodrug activation in vivo. In view of the quantitative relationship between CL intensity and CPT release established at the molecular level, the CL signal provides a real-time readout of activation extent and lays the groundwork for feedback-guided optimization of subsequent light dosing.

### 2.5 *In vivo* antitumor efficacy of CL-azo-CPT/Ce6 NPs

The antitumor activity of CL-Azo-CPT/Ce6 NPs was evaluated in a CT26-Luc1 peritoneal metastasis model. Seventy tumor-bearing BALB/c mice were randomly divided into seven groups (*n* = 10): Saline, Ce6 NPs, CL-Azo-CPT NPs, free CPT, CL-Azo-CPT/Ce6 NPs, Ce6 NPs + L, and CL-Azo-CPT/Ce6 NPs + L. The formulations were administered by intraperitoneal injection (100 μM, 250 μL) every 4 days for a total of four doses over 14 days, and the “L”-treated groups received white-light irradiation after each injection. Tumor progression was monitored by bioluminescence imaging (BLI) on days 0, 6, and 12 (Fig. 5A and B). The “Saline” and “Ce6 NPs” (without light) groups showed rapid tumor progression (bioluminescence signal enhancement), and all mice in these groups died before day 12. By contrast, “CL-Azo-CPT NPs”, “free CPT”, and “CL-Azo-CPT/Ce6 NPs” without irradiation delayed tumor growth to some extent. Both light-treated groups, “Ce6 NPs + L” and “CL-Azo-CPT/Ce6 NPs + L”, produced marked tumor suppression, with BLI signals on day 12 falling below the initial level. Body weight changes showed a similar trend. Most groups displayed progressive weight gain, consistent with ascites accumulation during peritoneal dissemination, whereas mice treated with “CL-Azo-CPT/Ce6 NPs + L” maintained relatively stable body weights (Fig. 5C).

**Fig. 5:**
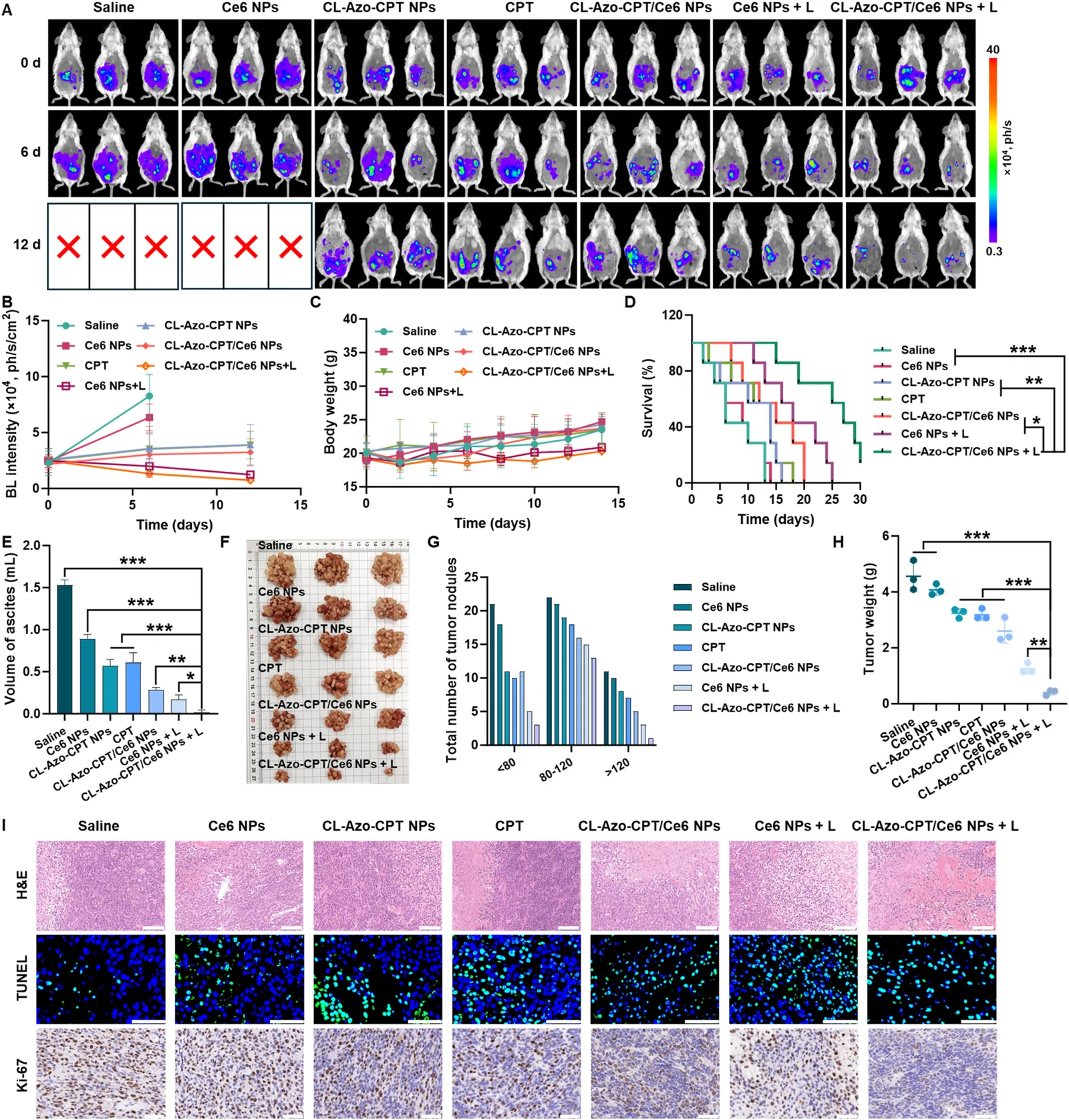
In vivo therapeutic efficacy in the CT26-Luc1 peritoneal carcinomatosis model. (A) Representative bioluminescence imaging (BLI) of mice on days 0, 6, and 12 (Exposure time: 10 s). (B) Corresponding quantification of whole-body BLI intensities. (C) Body weight variations during the treatment period. (D) Kaplan–Meier survival curves (*n* = 7). (E–H) Endpoint evaluation on day 14, including ascites volume (E), representative photographs of excised tumor nodules (F), nodule size distribution (G), and total weight of peri-gastric mesenteric tumors (H). (I) Representative H&E staining (scale bar = 100 μm), TUNEL immunofluorescence (scale bar = 50 μm), and Ki-67 immunohistochemistry (scale bar = 50 μm) of tumor sections harvested at the study endpoint. Data in (B, C, E, G, H) are presented as mean ± SD (*n* = 3). *P < 0.05, **P < 0.01, ***P < 0.001. Light-treated groups received abdominal light irradiation (0.8 W/cm^2^, 10 min) after each intraperitoneal injection.

Survival analysis further distinguished the treatment groups (Fig. 5D). Saline- and Ce6 NPs-treated mice all died within 15 days, whereas the chemotherapy-containing groups showed only limited survival extension and all animals in these groups died within 20 days. “Ce6 NPs + L” prolonged survival to approximately 25 days, while “CL-Azo-CPT/Ce6 NPs + L” gave the best outcome, with one mouse surviving beyond day 30. Together with the in vivo CL imaging results, these data support that light treatment enhances the therapeutic performance of the co-loaded formulation beyond that achieved by endogenous hypoxia alone.

Therapeutic efficacy was further assessed at the anatomical level on day 14. Ascites, perigastric nodules, and mesenteric nodules were collected after dissection. Macroscopic examination showed that the perigastric and mesenteric tissues from the “CL-Azo-CPT/Ce6 NPs + L” group were largely free of visible tumor nodules (Fig. S44). Quantitative analysis of ascites volume, tumor volume distribution, and total tumor weight was consistent with these observations (Fig. 5E–H). Notably, the tumor burden in the Ce6 NPs group was comparable to that in the Saline group, indicating negligible dark toxicity from the photosensitizer alone. Among all treatments, “CL-Azo-CPT/Ce6 NPs + L” gave the lowest tumor burden and the smallest ascites volume.

Histopathological analysis was in agreement with the gross findings. Hematoxylin and Eosin (H&E) staining of tumor tissues showed that both “Ce6 NPs + L” and “CL-Azo-CPT/Ce6 NPs + L” groups induced extensive necrotic regions relative to the “Saline”, “Ce6 NPs”, “CL-Azo-CPT NPs”, “free CPT”, and “CL-Azo-CPT/Ce6 NPs” groups, accompanied by tumor cell disintegration, eosinophilic cytoplasm, and nuclear pyknosis, karyorrhexis, and karyolysis (Fig. 5I). Terminal deoxynucleotidyl transferase dUTP nick end labeling (TUNEL) staining further showed that “CL-Azo-CPT/Ce6 NPs + L” produced the highest apoptotic index (72.81%), while Ki-67 staining revealed the lowest proliferative fraction, with only 2.84% positive cells (Fig. 5I and S45). These results indicate that although both light-treated groups produced substantial tissue damage, the co-loaded formulation achieved the strongest overall suppression of tumor cell survival and proliferation. Because Ce6 is excited at approximately 654 nm and light penetration in tissue is limited, this strategy is most suited to superficially accessible tumors or to settings in which light can be delivered locally, for example intraperitoneal lesions amenable to laparoscopic or intraoperative illumination, as modeled in the present peritoneal carcinomatosis study, as well as endoscopic-accessible lesions of the gastrointestinal tract.

The safety profile of the nanoplatform was also examined. Serum biochemical parameters, including hepatic and renal function markers, were assessed across all groups (Fig. S46). The levels of alanine aminotransferase (ALT) and urea (UREA) showed no significant differences between the healthy control and any of the tumor-bearing groups. However, tumor-bearing mice in the “Saline”, “Ce6 NPs”, “CL-Azo-CPT NPs”, “free CPT”, “CL-Azo-CPT/Ce6 NPs”, and “Ce6 NPs + L″ groups exhibited elevated aspartate aminotransferase (AST) levels and increased AST/ALT ratios compared with healthy mice, indicating that progressive peritoneal metastasis caused mild hepatic stress. Notably, the AST level and AST/ALT ratio of “CL-Azo-CPT/Ce6 NPs + L″ group were restored to levels comparable to those of the healthy control. In terms of renal function, except for slight fluctuations in creatinine (CREA) and uric acid (UA) levels in the Ce6 NPs group, all other treatment groups exhibited parameters similar to those of healthy mice. Furthermore, H&E staining of major organs showed that the mild pathological changes in the spleen and lung associated with advanced peritoneal carcinomatosis were largely alleviated in the “CL-Azo-CPT/Ce6 NPs + L” group, with no evident off-target tissue damage (Fig. S47). Together, these findings support the excellent biosafety of the integrated nanoplatform and suggest that effective tumor suppression concurrently mitigates tumor-induced systemic burden.

Although PDT-induced hypoxia amplification improves the activation of hypoxia-responsive prodrugs, excessive or sustained hypoxia can, in principle, promote malignant phenotypes, including enhanced invasiveness, metastasis, and local immunosuppression. The self-reporting design provides a way to address this concern. Because the chemiluminescence signal reports the extent of prodrug activation in real time, the light dose could in principle be titrated so that irradiation is stopped once the readout indicates adequate activation, thereby confining therapy to a “therapeutic window” that balances efficacy against the risk of hypoxia-driven tumor adaptation. Exploring such an adaptive dosing regimen, and defining the corresponding window experimentally, is an important direction for future work, and it is not yet realized in the present proof-of-concept formulation.

## 3 Conclusion

In summary, we have developed a self-reporting chemiluminescent nanoplatform (CL-Azo-CPT/Ce6 NPs) that establishes a “Sense-Amplify-Report” cascade. This system should be regarded as a feedback-guided and self-reporting platform rather than a fully closed-loop device. The real-time chemiluminescence readout can inform subsequent light-dosing decisions, while automated adjustment of the treatment in response to the signal remains a goal for future development. By rationally integrating a photosensitizer with an azobenzene-caged chemiluminescent prodrug, this system effectively circumvents the two fundamental bottlenecks of conventional hypoxia-activated prodrugs: inadequate endogenous hypoxia and the lack of in vivo activation feedback. Light irradiation drives local oxygen depletion to actively amplify tumor hypoxia, which in turn accelerates AZO-mediated prodrug cleavage. Crucially, this cleavage event strictly couples the release of the chemotherapeutic payload (CPT) with the emission of a tissue-penetrating chemiluminescent signal. Consequently, the luminescent readout provides a real-time, relative (semi-quantitative) spatiotemporal map of drug activation that can guide subsequent light-dosing decisions. The successful application of this adaptive strategy in a murine peritoneal metastasis model yielded profound tumor regression and prolonged survival, significantly outperforming passive monotherapies. Ultimately, this self-reporting, feedback-guiding theranostic paradigm offers a robust framework for designing next-generation precision nanomedicines capable of dynamically adapting to complex tumor microenvironments.

## CRediT authorship contribution statement

**Jie Gao:** Writing – review & editing, Writing – original draft, Methodology, Investigation, Funding acquisition, Data curation, Conceptualization. **Lianzhu Wei:** Writing – original draft, Investigation, Data curation. **Yujie Wang:** Validation, Methodology. **Maocheng Yang:** Validation, Investigation, Data curation. **Yumei Wu:** Writing – original draft, Validation, Funding acquisition. **Wen-Chao Geng:** Writing – review & editing, Supervision, Funding acquisition. **Zeli Yuan:** Writing – review & editing, Supervision, Funding acquisition, Conceptualization.

## Declaration of competing interests

The authors declare that they have no known competing financial interests or personal relationships that could have appeared to influence the work reported in this paper.

## Data Availability

Data will be made available on request.
