# Supplementary material for: Coupling photodynamic hypoxia amplification with a self-reporting chemiluminescent prodrug for adaptive precision therapy
Source: Mater Today Bio. 2026 Sep 24;41:103712. doi: 10.1016/j.mtbio.2026.103712 (PMC13635259; doi:10.1016/j.mtbio.2026.103712)
Supplement: Supplementary file 1 [file mmc1.docx]

**Supplementary Material**

**Coupling Photodynamic Hypoxia Amplification with a Self-Reporting Chemiluminescent Prodrug for Adaptive Precision Therapy**

Jie Gao,^a,#,^* Lianzhu Wei,^a,#^ Yujie Wang,^a^ Maocheng Yang,^a^ Yumei Wu,^a^ Wen-Chao Geng,^b,^* and Zeli Yuan^a,^*

^a^School of Pharmacy, Key Laboratory of Basic Pharmacology of Ministry of Education and Joint International Research Laboratory of Ethnomedicine of Ministry of Education, Guizhou International Science & Technology Cooperation Base of Medical Optical Theranostics Research, New Drug Discovery and Evaluation Center for International Cooperation and Disciplinary Innovation ("111 Center"), Zunyi Medical University, Zunyi, Guizhou 563003, P. R. China.

^b^College of Life Science and Technology, Beijing University of Chemical Technology, Beijing, 100029, China

^#^These authors contributed equally to this work.

*Corresponding authors.

E-mail: jiegao@zmu.edu.cn (J. Gao); wchgeng@buct.edu.cn (W.-C. Geng); zlyuan@zmu.edu.cn (Z. Yuan)

**1. Materials and Methods**

**1.1 Reagents and Instruments**

All chemicals and reagents were obtained from commercial suppliers and used without further purification unless otherwise noted. The chemiluminescent precursor, compound h, was synthesized according to a previously reported procedure.^[1]^

UV-vis absorption spectra were recorded on a Shimadzu UV-2600 spectrophotometer. Fluorescence spectra and kinetic profiles were acquired using an Agilent Cary Eclipse spectrophotometer and a SuPerMax 3100 plate reader, respectively. ^1^H and ^13^C NMR spectra were obtained on a Bruker Avance NEO 400 NMR Spectrometer. High-resolution mass spectrometry (HRMS) was performed on an Agilent 6550 Q-TOF instrument using electrospray ionization (ESI). High-performance liquid chromatography (HPLC) analyses were conducted on a Shimadzu system featuring an LC-40B XR solvent delivery unit, a DGU-403 degasser, a CTO-40S column oven, and an SPD-M40 photodiode array detector. Separation was achieved on a ZORBAX Eclipse Plus C18 column (3 × 150 mm, 3.5 μm). The particle size was measured using a 90Plus PALS high‑sensitivity zeta potential and particle size analyzer (Brookhaven Instruments Corporation, USA). Confocal fluorescence imaging was performed on a Leica STELLARIS 8 laser scanning confocal microscope. All *in vitro* and *in vivo* luminescence imaging was performed using a Berthold NightOWL II LB983 small animal imaging system.

**1.2 Synthesis**

**Synthesis of compound 2.** 4-Nitrobenzyl alcohol (6.0 g, 39.2 mmol) was dissolved in an aqueous NaOH solution (70 mL, 5.7 M) with heating. The solution was cooled to room temperature, and zinc powder (7.0 g, 107 mmol) was added in portions. The mixture was heated to reflux at 120 °C for approximately 10 h, then maintained at 70 °C for an additional 1 h. The resulting mixture was filtered, and the solid residue was washed thoroughly with deionized water and dried. The crude solid was suspended in hot methanol and filtered while hot to remove residual zinc powder. The filtrate was allowed to stand at room temperature, whereupon crystallization occurred. The crystals were collected by filtration and dried under vacuum to afford compound 2 as an orange crystalline solid (1.98 g, 21%). ^1^H NMR (400 MHz, DMSO-*d*_6_) δ 7.86 (d, *J* = 7.9 Hz, 4H), 7.52 (d, *J* = 8.0 Hz, 4H), 5.45 (t, *J* = 5.7 Hz, 2H), 4.60 (d, *J* = 6.0 Hz, 4H); ^13^C NMR (100 MHz, DMSO-*d*₆) δ 151.0, 146.4, 127.3, 122.6, 62.6.

**Synthesis of compound 3.** Camptothecin (CPT, 174 mg, 0.50 mmol) was dissolved in dichloromethane (10 mL) and cooled to 0 °C. A solution of DMAP (183 mg, 1.50 mmol) in dichloromethane was added rapidly, followed by dropwise addition of a solution of triphosgene (47 mg, 0.16 mmol) in dichloromethane. The mixture was stirred at 0 °C for 10 min, then warmed to room temperature. A solution of compound 2 (121 mg, 0.50 mmol) in dichloromethane was added dropwise, and the reaction was monitored by TLC. Upon completion, the mixture was diluted with dichloromethane, washed with deionized water, and the organic phase was dried over anhydrous Na_2_SO_4_. The solvent was removed under reduced pressure, and the crude product was purified by silica gel column chromatography (DCM/MeOH = 25:1, v/v) to afford compound 2 as an orange solid (103 mg, 33%). ^1^H NMR (400 MHz, CDCl_3_/CD_3_OD) δ 8.22 (d, *J* = 14.2 Hz, 1H), 8.06 (d, *J* = 8.5 Hz, 1H), 7.87 (d, *J* = 8.7 Hz, 1H), 7.74 (d, *J* = 8.2 Hz, 1H), 7.69–7.55 (m, 4H), 7.53–7.47 (m, 1H), 7.37–7.27 (m, 4H), 7.23 (s, 1H), 5.57 (d, *J* = 17.1 Hz, 1H), 5.26 (d, *J* = 17.0 Hz, 1H), 5.12–5.01 (m, 4H), 4.60 (d, *J* = 11.8 Hz, 2H), 2.20–1.97 (m, 2H), 0.91 (t, *J* = 7.5 Hz, 3H); ^13^C NMR (100 MHz, CDCl_3_/CD_3_OD) δ 167.5, 157.3, 153.4, 152.2, 151.7, 151.4, 148.5, 146.3, 146.0, 144.6, 137.1, 131.4, 130.8, 129.2, 128.6, 128.2, 128.1, 127.1, 125.6, 122.8, 122.1, 120.6, 119.7, 96.2, 78.0, 69.7, 66.8, 63.9, 49.9, 31.5, 7.5.

**Synthesis of compound 4.** The compound h (29 mg, 0.081 mmol) and triphosgene were dissolved in anhydrous dichloromethane (5 mL) and cooled to 0 °C. Pyridine was added dropwise, and the mixture was stirred at 0 °C for 1 h, then at room temperature for 2 h. The solvent was removed under reduced pressure at room temperature. A solution of compound 3 (50 mg, 0.081 mmol) in dichloromethane (5 mL) was added dropwise to the residue, and the reaction was stirred at room temperature for 5 h, monitored by TLC. Upon completion, the mixture was diluted with dichloromethane, washed with deionized water, and the organic phase was dried over anhydrous Na_2_SO_4_. The solvent was removed under reduced pressure, and the crude product was purified by silica gel column chromatography (DCM/MeOH = 25:1, v/v) to afford compound 4 as an orange solid (35 mg, 43%). ^1^H NMR (400 MHz, CDCl₃) δ 8.61 (s, 1H), 8.34 (d, *J* = 8.8 Hz, 1H), 8.21 (d, *J* = 8.4 Hz, 1H), 7.88 (d, *J* = 8.2 Hz, 1H), 7.82–7.79 (m, 2H), 7.71–7.59 (m, 2H), 7.56 (d, *J* = 8.3 Hz, 1H), 7.50–7.43 (m, 3H), 7.32–7.25 (m, 4H), 5.99 (d, *J* = 16.7 Hz, 1H), 5.71 (d, *J* = 17.2 Hz, 1H), 5.47–5.33 (m, 3H), 5.32–5.24 (m, 3H), 5.20 (d, *J* = 11.4 Hz, 2H), 3.30 (s, 3H), 3.26 (s, 1H), 2.29–2.15 (m, 2H), 2.07 (s, 1H), 1.97–1.74 (m, 12H), 1.02 (t, *J* = 7.5 Hz, 3H); ^13^C NMR (100 MHz, CDCl_3_) δ 167.5, 157.4, 153.6, 152.6, 152.4, 152.3, 151.9, 149.9, 149.0, 146.6, 145.9, 143.3, 139.3, 138.6, 137.7, 137.1, 136.1, 134.1, 131.3, 130.9, 129.8, 129.0, 128.5, 128.3, 128.2, 127.8, 126.1, 126.0, 124.6, 123.4, 123.3, 122.9, 120.3, 117.6, 100.4, 96.0, 78.2, 70.9, 69.9, 67.2, 57.6, 50.1, 38.7, 37.0, 33.0, 31.9, 29.9, 28.3, 7.8; HR-MS (ESI): *m/z* calcd for C_57_H_48_ClN_5_NaO_10_^+^ [M + Na]^+^ 1020.2982, found 1020.2994.

**Synthesis of CL-Azo-CPT.** Compound 4 (100 mg, 0.10 mmol) was dissolved in dichloromethane (20 mL), and a catalytic amount of methylene blue was added as a photosensitizer. Oxygen was bubbled through the solution under red-light irradiation for 1 h, and the reaction was monitored by TLC. Upon completion, the solvent was removed under reduced pressure. The crude product was passed through a silica gel column to remove methylene blue, followed by purification via preparative HPLC (MeCN/H_2_O = 90:10, v/v) to afford CL-Azo-CPT as an orange solid (34 mg, 33%). ^1^H NMR (400 MHz, CDCl_3_) δ 8.33 (s, 1H), 8.21 (d, *J* = 8.5 Hz, 1H), 8.09 (d, *J* = 8.4 Hz, 1H), 7.88 (d, *J* = 8.2 Hz, 1H), 7.81 (t, *J* = 7.4 Hz, 5H), 7.64 (d, *J* = 6.9 Hz, 1H), 7.59–7.53 (m, 3H), 7.51–7.47 (m, 2H), 7.30 (s, 1H), 6.06 (d, *J* = 16.7 Hz, 1H), 5.71 (d, *J* = 17.1 Hz, 1H), 5.41 (s, 3H), 5.36 (s, 1H), 5.26 (d, *J* = 3.7 Hz, 2H), 5.21 (s, 2H), 3.19 (s, 3H), 3.00 (s, 1H), 2.32–2.13 (m, 3H), 1.95–1.54 (m, 12H), 1.02 (t, *J* = 7.5 Hz, 3H); ^13^C NMR (100 MHz, CDCl_3_) δ 167.5, 157.4, 153.7, 152.7, 152.4, 152.3, 151.8, 149.0, 146.6, 145.8, 142.8, 137.8, 137.0, 136.5, 131.3, 130.8, 129.8, 129.6, 129.1, 128.9, 128.5, 128.3, 128.2, 127.5, 124.8, 123.4, 123.3, 120.3, 117.3, 111.5, 101.8, 96.5, 96.0, 95.9, 78.2, 71.0, 69.9, 67.1, 50.1, 36.6, 34.0, 33.7, 32.5, 32.2, 31.9, 31.6, 26.2, 25.8, 7.8; HR-MS (ESI): *m/z* calcd for C_57_H_48_ClN_5_NaO_12_^+^ [M + Na]^+^ 1052.2880, found 1052.2896.

Scheme S1 Synthetic route for the CL-Azo-CL.

**Synthesis of Synthesis of compound 3a.** The compound h (356 mg, 1.00 mmol) and triphosgene were dissolved in anhydrous dichloromethane (10 mL) and cooled to 0 °C. Pyridine was added dropwise, and the mixture was stirred at 0 °C for 1 h, then at room temperature for 2 h. The solvent was removed under reduced pressure at room temperature. A solution of compound 2 (121 mg, 0.50 mmol) in dichloromethane (5 mL) was added dropwise to the residue, and the reaction was stirred at room temperature for 5 h, monitored by TLC. Upon completion, the mixture was diluted with dichloromethane, washed with deionized water, and the organic phase was dried over anhydrous Na_2_SO_4_. The solvent was removed under reduced pressure, and the crude product was purified by silica gel column chromatography (PE/EA = 3:1, v/v) to afford compound 3a as an orange solid (331 mg, 66%). ^1^H NMR (400 MHz, CDCl_3_) δ 7.98 (d, *J* = 8.4 Hz, 4H), 7.60 (d, *J* = 8.4 Hz, 4H), 7.50 (d, *J* = 16.7 Hz, 2H), 7.44 (d, *J* = 8.1 Hz, 2H), 7.26 (d, *J* = 8.2 Hz, 2H), 5.98 (d, *J* = 16.7 Hz, 2H), 5.42 (s, 4H), 3.30 (s, 6H), 3.27 (s, 2H), 2.05 (s, 2H), 1.97–1.75 (m, 24H); ^13^C NMR (100 MHz, CDCl_3_) δ 152.7, 151.7, 145.6, 143.2, 139.2, 138.5, 137.1, 133.9, 129.4, 128.9, 127.6, 124.5, 123.4, 117.5, 100.4, 70.7, 60.4, 57.5, 38.5, 36.9, 32.9, 29.7, 28.2.

**Synthesis of CL-Azo-CL.** Compound 3a (101 mg, 0.10 mmol) was dissolved in dichloromethane (20 mL), and a catalytic amount of methylene blue was added as a photosensitizer. Oxygen was bubbled through the solution under red-light irradiation for 1 h, and the reaction was monitored by TLC. Upon completion, the solvent was removed under reduced pressure. The crude product was passed through a silica gel column to remove methylene blue, followed by purification via preparative HPLC (MeCN/H_2_O = 90:10, v/v) to afford CL-Azo-CL as an orange solid (69 mg, 64%). ^1^H NMR (400 MHz, CDCl_3_) δ 8.10 (d, *J* = 8.4 Hz, 2H), 7.99 (d, *J* = 7.9 Hz, 4H), 7.60 (t, *J* = 8.1 Hz, 6H), 7.53 (s, 1H), 7.51 (s, 1H), 6.06 (d, *J* = 16.7 Hz, 2H), 5.42 (s, 4H), 3.20 (s, 6H), 3.01 (s, 2H), 1.94 (s, 2H), 1.88–1.47 (m, 24H); ^13^C NMR (100 MHz, CDCl_3_) δ 152.8, 151.8, 146.2, 142.9, 142.7, 137.1, 136.5, 129.6, 129.2, 127.5, 123.5, 117.3, 111.5, 101.9, 101.7, 96.5, 71.0, 50.0, 36.6, 33.7, 32.2, 31.5, 26.1; HR-MS (ESI): *m/z* calcd for C_58_H_54_Cl_2_N_4_NaO_12_^+^ [M + Na]^+^ 1091.3007, found 1091.3004.

**1.3 Chemiluminescence Kinetics Measurements**

Tris-HCl buffer solutions (10 mM, pH 7.4, 1% DMSO) containing 10 μM CL-Azo-CPT or CL-Azo-CL were added to 96-well plates. Following the addition of Na₂S₂O₄ (final concentration: 5 mM), the chemiluminescence (CL) intensity was monitored continuously over 8 h at 37 °C using a multifunctional microplate reader. The kinetics without Na_2_S_2_O_4_ were also recorded for comparison. The reaction rate constant and half-life were calculated using Equation 1:

$I(t)=I_{\text{max}}e^{-k_{fall}t}$ Equation 1

Where *I*(*t*) represents the real-time chemiluminescence intensity, *I*_max_ is the maximum chemiluminescence intensity, *k*_f_ is the decay rate constant, and *t* is the reaction time.

**1.4 Limit of Detection (LOD)**

The linear relationship between the luminescence intensity of the probes and Na_2_S_2_O_4_ concentration was established. Tris-HCl buffer containing 10 μM CL-Azo-CPT or CL-Azo-CL was added to a 96-well plate, followed by the addition of diluted Na_2_S_2_O_4_ stock solutions to final concentrations of 0, 0.2, 0.4, 0.6, and 0.8 mM. The luminescence intensity was measured at 37 °C using a microplate reader.

The background luminescence of the probes was measured 11 times to obtain the standard deviation (*σ*) of the blank. Based on the slope (*k*) of the linear fit between luminescence intensity and Na_2_S_2_O_4_ concentration, the LOD was calculated using Equation 2:

$LOD=3\sigma/k$ Equation 2

**1.5 Selectivity Study**

The interference from potential physiological substances (reactive oxygen species, amino acids, common ions, and enzymes) was investigated. Tris-HCl buffer solutions containing 10 μM sample were incubated with the following analytes at 37 °C: H_2_O_2_ (10 μM), cysteine (Cys, 1 mM), threonine (Thr, 1 mM), valine (Val, 1 mM), dithiothreitol (DTT, 1 mM), vitamin C (1 mM), glucose (10 mM), glutathione (GSH, 5 mM), Na^+^ (150 mM), K+ (150 mM), Mg^2+^ (2.5 mM), Ca^2+^ (2.5 mM each), pepsin (100 μg/L), trypsin (200 ng/mL), and Na_2_S_2_O_4_ (0.8 mM). Chemiluminescence intensities were measured using a multimode microplate reader.

**1.6 Drug Release Studies**

CL-Azo-CPT or CL-Azo-CL was incubated with Na_2_S_2_O_4_ (final concentration 40 mM) in Tris-HCl buffer at 37 °C with shaking. At predetermined time points guided by the chemiluminescence kinetics, aliquots were analyzed by high-performance liquid chromatography (HPLC) to monitor the release of CPT and the CL product MCCH. Chemiluminescence intensities at varying Na_2_S_2_O_4_ concentrations were also measured and plotted.

HPLC elution conditions: 40% A for 2 min, followed by a gradient from 40% to 80% A over 10 min, and then 80% to 95% A over 10 min; mobile phase A: acetonitrile (0.1% TFA), mobile phase B: water (0.1% TFA).

**1.7 Preparation of Nanoparticles**

CL-Azo-CPT NPs, CL-Azo-CL NPs, Ce6 NPs, and CL-Azo-CPT/Ce6 NPs were prepared using a thin-film hydration method. Appropriate amounts of the respective components and DSPE-mPEG_2000_ were weighed and dissolved in 1 mL of tetrahydrofuran (THF). The molar ratios were designed as follows: DSPE-mPEG_2000_ : CL-Azo-CPT = 8:1; DSPE-mPEG_2000_ : CL-Azo-CL = 8:1; DSPE-mPEG_2000_ : Ce6 = 8:1; and DSPE-mPEG_2000_ : CL-Azo-CPT:Ce6 = 8:1:1. The solvent was removed by rotary evaporation at 25 °C and 60 rpm to form a thin film, followed by further drying using a diaphragm pump and a vacuum oven for 12 h. The dried film was hydrated with 1 mL of Tris-HCl buffer (10 mM, pH 7.4) and subjected to sonication for 5 min (40% amplitude) in an ice bath to obtain the final nanoparticle solutions (1 mM).

**1.8 Determination of Encapsulation Efficiency, Drug Loading Capacity, and Particle Size**

The prepared nanoparticles were separated from unencapsulated free drugs by ultrafiltration to determine the encapsulation efficiency of CL‑Azo‑CPT and Ce6 in drug‑loaded nanoparticles.^[2]^ Freshly prepared CL‑Azo‑CPT/Ce6 NPs were loaded into the upper chamber of an ultrafiltration centrifuge tube (MWCO 100 kDa, Millipore Amicon Ultra, Merck, Germany) and centrifuged at 3000 rpm for 10 min using a low‑speed centrifuge. The filtrate collected from the lower chamber of the ultrafiltration tube was analyzed via high‑performance liquid chromatography (HPLC). Gradient: 45% B to 100% B for 20 min; A: water (containing 0.1% TFA), B: MeCN (containing 0.1% TFA).

The mass of free drug, denoted as *W*_1_, was calculated according to the standard curve. An aliquot of freshly prepared CL‑Azo‑CPT/Ce6 NPs dispersion was demulsified by sonication for 10 min after adding absolute ethanol, and then subjected to HPLC measurement. The total mass of drug, denoted as *W*_2_, was obtained using the corresponding standard curve. The encapsulation efficiency (EE) was calculated by the following formula:

$EE(\%)=\frac{W_{2}-W_{1}}{W_{2}}\times100\%$ Equation 3

The drug‑loading capacity (DLC) of nanoparticles was further calculated using the equation:

$DLC(\%)=\frac{W_{2}-W_{1}}{(W_{2}-W_{1})+W_{3}}\times100\%$ Equation 4

where *W*_3_ is the total weight of DSPE‑mPEG2000 material.

The prepared NP stock solutions (1 mM) were diluted to 100 μM with Tris-HCl buffer (10 mM, pH 7.4). The hydrodynamic diameters of CL-Azo-CPT NPs, Ce6 NPs, and CL-Azo-CPT/Ce6 NPs were measured by dynamic light scattering (DLS). Their morphologies were observed by transmission electron microscopy (TEM). The size stability of CL-Azo-CPT/Ce6 NPs was monitored over 7 days.

**1.9 Singlet Oxygen Detection**

9,10-Anthracenediyl-bis(methylene)dimalonic acid (ABDA) was used as an ^1^O_2_ indicator. Stock solutions of ABDA and Ce6 (1 mM) were prepared in DMSO. Various groups were prepared in Tris-HCl buffer (10 mM, pH 7.4) in quartz cuvettes: ABDA alone (50 μM); free Ce6 (10 μM) + ABDA (50 μM); Ce6 NPs (10 μM Ce6 equiv.) + ABDA (50 μM); and CL-Azo-CPT/Ce6 NPs (10 μM Ce6 equiv.) + ABDA (50 μM). The solutions were saturated with oxygen for 10 s prior to each irradiation. White light irradiation was applied for 1 min per cycle for a total of 15 min. The absorbance spectra (200–800 nm) were recorded at 25 °C using a UV-vis spectrophotometer. The normalized absorbance changes of ABDA at 402 nm were plotted against irradiation time.

**1.10 Cell Experiments**

**1.10.1 Cell Culture**

Human colorectal carcinoma cells (HCT116) and mouse colorectal carcinoma cells (CT26) were obtained from Zhejiang Meisen Cell Technology Co., Ltd. Luciferase-expressing mouse colorectal carcinoma cells (CT26-Luc1) were also purchased from Zhejiang Meisen Cell Technology Co., Ltd. All cells were cultured in DMEM/1640 medium supplemented with 10% fetal bovine serum (FBS) and 1% penicillin-streptomycin in a humidified incubator at 37 °C with 5% CO_2_. Cells in the logarithmic growth phase were used for experiments.

**1.10.2 Cell Viability Assay**

**Screening of CoCl_2_ concentration.** CT26 and HCT116 cells were seeded into 96-well plates at a density of 5,000 cells/well and incubated for 16 h. CoCl_2_ was then added at various concentrations (0–1050 μM), and cells were incubated for an additional 8 h to mimic hypoxia. The medium was replaced with fresh medium, and the cells were incubated for another 24 h. Afterward, 20 μL of MTT solution (5 mg/mL) was added to each well, followed by 4 h of incubation. The medium was carefully removed, and the formed formazan crystals were dissolved in 100 μL of DMSO per well. The absorbance at 490 nm was measured using a microplate reader to calculate cell viability and determine the optimal CoCl_2_ concentration.

**Cytotoxicity assay.** Cells were seeded in 96-well plates (5,000 cells/well). For the normoxia group, cells were incubated for 24 h. For the hypoxia group, cells were incubated for 16 h, followed by the addition of CoCl_2_ and an 8 h incubation. The medium was then replaced with fresh medium containing different concentrations (0–16 μM) of free CPT, CL-Azo-CPT NPs, CL-Azo-CL NPs, Ce6 NPs, or CL-Azo-CPT/Ce6 NPs. For dark toxicity, cells were continuously incubated for 24 h. For phototoxicity, cells were incubated for 6 h, irradiated with white light (220.7 mW/cm^2^) for 30 s, and then incubated for a further 18 h. Cell viability was determined using the MTT assay as described above. Viability was calculated as: [OD(experimental) – OD(blank)] / [OD(control) – OD(blank)] × 100%.

**1.10.3 Live/Dead Cell Staining**

HCT116 and CT26 cells (~1 × 10^5^) were seeded into confocal dishes. After establishing normoxic or hypoxic conditions (using CoCl_2_ as described above), cells were incubated with fresh medium containing CL-Azo-CPT NPs, Ce6 NPs, or CL-Azo-CPT/Ce6 NPs. The dark groups were incubated for 12 h. The light irradiation groups were incubated for 6 h, exposed to white light (220.7 mW/cm²) for 30 s, and further incubated for 6 h. After washing, cells were co-stained with Calcein-AM/PI (v/v = 1:1) for 30 min and observed under an inverted fluorescence microscope.

**1.10.4 Fluorescence Imaging In Vitro**

HCT116 and CT26 cells (~1 × 10^5^) were seeded into confocal dishes and incubated for 24 h. Cells were then exposed to fresh medium for 6 h (normoxia) or CoCl_2_-containing medium for 2, 4, or 6 h (hypoxia). After washing twice with PBS, the cells were incubated with CL-Azo-CPT/Ce6 NPs (10 μM) for 6 h. Intracellular fluorescence was detected using confocal laser scanning microscopy (CLSM). Imaging conditions: CPT (*λ*_ex_ = 405 nm, *λ*_em_ = 415–480 nm); CL (*λ*_ex_ = 490 nm, *λ*_em_ = 510–650 nm); Ce6 (*λ*_ex_ = 665 nm, *λ*_em_ = 672–850 nm).

**1.10.5 Chemiluminescence Imaging In Vitro**

**Hypoxia-dependent activation.** CT26 and HCT116 cells (5,000 cells/well) were seeded into 96-well plates. After pretreatment under normoxic (6 h) or hypoxic (CoCl₂ for 2, 4, or 6 h) conditions, cells were incubated with CL-Azo-CPT/Ce6 NPs (20 μM) for 2 h. After washing with PBS, chemiluminescence was recorded using an in vivo imaging system (IVIS) with an exposure time of 60 s.

**Concentration dependence.** Following the aforementioned normoxic or hypoxic (6 h) pretreatments, cells were incubated with varied concentrations of CL-Azo-CPT/Ce6 NPs (10, 20, 30, 40, and 50 μM) for 2 h. Chemiluminescence images were acquired via IVIS (60 s exposure).

**1.10.6 Intracellular ROS Detection**

HCT116 and CT26 cells (5×10^4^ cells/well) were seeded in 12-well plates and subjected to normoxic or hypoxic (CoCl_2_ treatment) conditions.

Light group: Cells were incubated with CL-Azo-CPT NPs, CL-Azo-CPT/Ce6 NPs, or Ce6 NPs (10 µM) for 6 h. After replacing the medium, DCFH-DA (10 μM) was added and incubated for 30 min. Cells were washed with PBS, irradiated with white light (220.7 mW/cm^2^) for 0.5 min, and imaged using an inverted fluorescence microscope.

Dark group: Same as the light group but without irradiation.

Control groups: Cells were incubated with DCFH-DA (10 μM) alone, with or without white light irradiation, and imaged under identical conditions.

**1.11 Animal Experiments**

**1.11.1 Animal Care**

All animal experiments were strictly performed in compliance with the *Guide for the Care and Use of Laboratory Animals* and were approved by the Ethics Committee of Zunyi Medical University (Ethics No.: ZMU21-2403-047). Animals were housed in grouped cages (*n* = 5–7 per cage) with good ventilation, a constant temperature of 23 ± 1 °C, and 55 ± 5% humidity. All mice were provided with a standard laboratory diet and water *ad libitum*.

**1.11.2 In Vivo Chemiluminescence and Fluorescence Imaging**

Female BALB/cAn-nu mice (5 weeks old, 15–18 g) were purchased from Beijing Vital River Laboratory Animal Technology Co., Ltd. (SPF grade, License No.: SCXK (Jing) 2023-0004). To establish the peritoneal metastasis model of colorectal cancer, mice were intraperitoneally (i.p.) injected with 200 μL of CT26-Luc1 cells (1×10^7^ cells/mL). Tumor progression was monitored using an IVIS by i.p. injection of diphenylterazine (DTZ); subsequent experiments began when the bioluminescence signal reached 1× 10^5^ ph/s/cm^2^. For chemiluminescence imaging, CL-Azo-CPT NPs or CL-Azo-CPT/Ce6 NPs (100 μM, 200 μL) were i.p. injected, and images were captured under bioluminescence mode (60 s exposure). For *in vivo* fluorescence imaging, mice were anesthetized and i.p. injected with CL-Azo-CPT/Ce6 NPs (100 μM, 200 μL), followed by signal acquisition using the IVIS.

**1.11.3 Antitumor Efficacy**

Female BALB/c mice (5–6 weeks old, 16–22 g) were purchased from the Experimental Animal Center of Zunyi Medical University (SPF grade, License No.: SCXK (Qian) 2021-0002). After shaving their abdomens, mice were i.p. injected with CT26-Luc1 cells (200 μL, 1×10^7^ cells/mL). Upon tumor bioluminescence reaching 1× 10^5^ (via DTZ detection), 70 tumor-bearing mice were randomly divided into 7 groups (*n* = 10 per group): (1) Saline, (2) Ce6 NPs, (3) CL-Azo-CPT NPs, (4) CPT, (5) CL-Azo-CPT/Ce6 NPs, (6) Ce6 NPs + Light, and (7) CL-Azo-CPT/Ce6 NPs + Light. All light treatments were performed with white light irradiation at a power density of 0.8 W/cm^2^ for 10 min.

The initial body weights were recorded. Mice received i.p. injections of the corresponding formulations (100 μM, 250 μL) every 4 days for a total of 4 treatments over 13 days. Body weights were measured every other day for 14 days (7 times total). Therapeutic efficacy was monitored concurrently via chemiluminescence imaging across the 7 groups. A 30-day survival analysis was also initiated. On day 14, three mice from each group were randomly selected, euthanized, and dissected to assess biosafety; intraperitoneal ascites volume was collected and compared among groups.

**1.11.4 Histological Analysis**

At the end of the treatment period, blood samples were collected to assess hepatic function markers, including aspartate aminotransferase (AST) and alanine aminotransferase (ALT), and renal function markers, including creatinine (Crea), uric acid (UA), urea (UREA). The results were compared with those of the saline group (*n* = 3). Major organs, including the heart, liver, spleen, lungs, and kidneys, were harvested, fixed in 4% paraformaldehyde, embedded in paraffin, sectioned, and stained with hematoxylin and eosin (H&E) for histopathological evaluation (*n* = 3 per group). Tumor tissues were processed similarly and subjected to H&E staining, terminal deoxynucleotidyl transferase dUTP nick-end labeling (TUNEL) fluorescence staining, and Ki67 immunohistochemistry to evaluate tumor cell damage, apoptosis, and proliferation.

**1.11.5 Statistical Analysis**

All quantitative data are expressed as mean ± standard deviation (SD). Differences between experimental and control groups were analyzed using One-way ANOVA and Student’s *t* test. Survival data are presented as Kaplan-Meier curves and analyzed by the Gehan-Breslow-Wilcoxon test. Statistical significance is denoted as **P* < 0.05, ***P* < 0.01, and ****P* < 0.001.

**2. Structural Characterization**

Figure S1 ^1^H NMR spectrum of Compound 2 in DMSO-*d*_6_ (400 MHz, 298 K).

Figure S2 ^13^C NMR spectrum of Compound 2 in DMSO-*d*_6_ (100 MHz, 298 K).

Figure S3 ^1^H NMR spectrum of Compound 3 in CDCl_3_/CD_3_OD (400 MHz, 298 K).

Figure S4 ^13^C NMR spectrum of Compound 3 in CDCl_3_/CD_3_OD (100 MHz, 298 K).

Figure S5 ^1^H NMR spectrum of Compound 4 in CDCl_3_ (400 MHz, 298 K).

Figure S6 ^13^C NMR spectrum of Compound 4 in CDCl_3_ (100 MHz, 298 K).

Figure S7 ^1^H NMR spectrum of Compound CL-Azo-CPT in CDCl_3_ (400 MHz, 298 K).

Figure S8 ^13^C NMR spectrum of Compound CL-Azo-CPT in CDCl_3_ (100 MHz, 298 K).

Figure S9 HRMS (ESI-MS) spectrum of Compound 4.

Figure S10 HRMS (ESI-MS) spectrum of CL-Azo-CPT.

Figure S11 ^1^H NMR spectrum of Compound 3a in CDCl_3_ (400 MHz, 298 K).

Figure S12 ^13^C NMR spectrum of Compound 3a in CDCl_3_ (100 MHz, 298 K).

Figure S13 ^1^H NMR spectrum of Compound CL-Azo-CL in CDCl_3_ (400 MHz, 298 K).

Figure S14 ^13^C NMR spectrum of Compound CL-Azo-CL in CDCl_3_ (100 MHz, 298 K).

Figure S15 HRMS (ESI-MS) spectrum of CL-Azo-CL.

Figure S16 RP-HPLC chromatograms of CL-Azo-CPT. Gradient: 40% A for 2 min, then 40% A to 80% A for 10 min, finally 80% A to 95% A for 10 min; A: MeCN (containing 0.1% TFA), B: water (containing 0.1% TFA).

**3. Supporting Results**

Figure S17 (A) Chemiluminescence spectra of prodrug CL-Azo-CL (10 μM) in the presence and absence of Na_2_S_2_O_4_ (5 mM). (B) Chemiluminescence kinetics curves of CL-Azo-CL (10 μM) in Tris-HCl (pH=7.4) in the presence and absence of Na_2_S_2_O_4_ (5 mM).

Figure S18 Chemiluminescence kinetic profiles of various concentrations of prodrug CL-Azo-CPT (2, 4, 6, 8, and 10 μM) in Tris-HCl (pH=7.4) in the presence of Na_2_S_2_O_4_ (0.8 mM).

Figure S19 (A) Linear correlation between chemiluminescence intensity of CL-Azo-CL and the corresponding concentration of Na_2_S_2_O_4_ (mean ± SD, *n* = 3). (B) Chemiluminescence response of prodrug CL-Azo-CL (10 µM) to various reactive analytes and enzymes (mean ± SD, *n* = 3).

Figure S20 RP-HPLC chromatograms in the presence of Na_2_S_2_O_4_ (0–40 mM). Gradient: 40% A for 2 min, then 40% A to 80% A for 10 min, finally 80% A to 95% A for 10 min; A: MeCN (containing 0.1% TFA), B: water (containing 0.1% TFA).

Figure S21 The Fluorescence spectra of (A) CL-Azo-CPT (10 μM) or (B) CL-Azo-CL in the presence and absence of Na_2_S_2_O_4_ were recorded (*λ*_ex_ = 420 nm).

Figure S22 DLS analysis of CL-Azo-CPT NPs (A), Ce6 NPs (B), and CL-Azo-CL NPs.

Figure S23 TEM images of CL-Azo-CPT NPs and Ce6 NPs. Scale bar: 1 μm

Figure S24 (A) The UV absorption spectra of Ce6 and CL-Azo-CPT/Ce6 NPs (10 μM) were recorded. (B) The Fluorescence spectra of Ce6 and CL-Azo-CPT/Ce6 NPs (10 μM) were recorded.

Figure S25 ^1^O_2_ generation capabilities under white light irradiation. (A–C) Time-dependent absorption spectra of (A) ABDA alone (50 μM), (B) free Ce6 (10 μM) with ABDA (50 μM), and (C) Ce6 NPs (10 μM) with ABDA (50 μM) in Tris-HCl buffer (10 mM, pH 7.4) upon white light irradiation (0.8 W cm^–2^) for 15 min. (D) Normalized absorbance decay of ABDA at 402 nm for different formulations (ABDA alone, free Ce6, Ce6 NPs, and CL-Azo-CPT/Ce6 NPs).

Figure S26 Cell viability of (a) CT26 or (b) HCT116 cells after CoCl_2_ treatments (mean ± SD, *n* = 6).

Figure S27 (A) CLSM fluorescence images of CT26 cells treated with CL-Azo-CPT/Ce6 NPs under hypoxia for 2, 4, or 6 h (or under normoxia for 6 h). Scale bar: 20 μm. (B) Quantitative results of (A) (mean ± SD, *n* = 3; ns, no significance; **P* < 0.05; ***P* < 0.01.

Figure S28 (A) CLSM fluorescence images of HCT116 cells treated with CL-Azo-CPT/Ce6 NPs under hypoxia for 2, 4, or 6 h (or under normoxia for 6 h). Scale bar: 20 μm. (B) Quantitative results of (A) (mean ± SD, *n* = 3; ns, no significance; **P* < 0.05; ***P* < 0.01, ****P* < 0.001).

Figure S29 Chemiluminescence intensity of CL-Azo-CPT/Ce6 NPs (20 μM) in CT26 (A) and HCT116 (B) cells under different hypoxia time (mean ± SD, *n* = 3).

Figure S30 Chemiluminescence intensity of CT26 (A) and HCT116 (B) cells with different concentrations of CL-Azo-CPT/Ce6 NPs under hypoxic conditions. Exposure time: 60 s (mean ± SD, *n* = 3).

Figure S31 Fluorescence images of ROS in CT26 cells after various treatments, imaged by fluorescence microscopy with DCFH-DA as ROS fluorescence probe. Scale bar: 200 μm. For light-treated groups, cells were irradiated with white light (220.7 mW/cm^2^) for 30 s.

Figure S32 Fluorescence images of ROS level in HCT-116 cells after various treatments, imaged by fluorescence microscopy using DCFH-DA as ROS fluorescence probe. Scale bar: 200 μm. For light-treated groups, cells were irradiated with white light (220.7 mW/cm^2^) for 30 s.

Figure S33 Cell viability (A, B) and IC_50_ values (C, D) of CT26 or HCT116 cells after free CPT treatments under normoxic or hypoxic condition (mean ± SD, *n* = 6).

Figure S34 Cell viability of CT26 or HCT116 cells after CL-Azo-CL NPs treatments under normoxic or hypoxic condition (mean ± SD, *n* = 6).

Figure S35 Cell viability of Ce6 NPs, CL-Azo-CPT NPs and CL-Azo-CPT/Ce6 NPs against CT26 cells in normoxic (A, B) or hypoxic (C, D) condition under light irradiation (220.7 mW/cm^2^, 30 s) or in dark (mean ± SD, *n* = 6).

Figure S36 Cell viability of Ce6 NPs, CL-Azo-CPT NPs and CL-Azo-CPT/Ce6 NPs against HCT116 cells in normoxic (A, B) or hypoxic (C, D) condition under light irradiation (220.7 mW/cm^2^, 30 s) or in dark (mean ± SD, *n* = 6).

Figure S37 The IC_50_ values of Ce6 NPs, CL-Azo-CPT NPs, and CL-Azo-CPT/Ce6 NPs against CT26 (A–C) and HCT116 (D–F) cells in different conditions. (mean ± SD, *n* = 6). For light-treated groups, cells were irradiated with white light (220.7 mW/cm^2^) for 30 s.

Table 1. IC50 values (μM) of different formulations against CT26 and HCT116 cells under various conditions. ^[a]^

| **Formulation** | **Cell Line** | | **Normoxia (Dark)** | **Normoxia (Light)** | **Hypoxia (Dark)** | **Hypoxia (Light)** |
| --- | --- | --- | --- | --- | --- | --- |
| Free CPT | CT26 | 4.7 | | – | 7.9 | – |
|  | HCT116 | 5.9 | | – | 3.2 | – |
| Ce6 NPs | CT26 | >> 16^[b]^ | | 6.2 | >> 16 | 8.8 |
|  | HCT116 | >> 16 | | 4.4 | >> 16 | 7.3 |
| CL-Azo-CPT NPs | CT26 | >> 16 | | >> 16 | 17.5 | 16.7 |
|  | HCT116 | >> 16 | | >> 16 | 14.6 | 11.9 |
| CL-Azo-CPT/Ce6 NPs | CT26 | >> 16 | | 4.2 | 8.2 | 3.0 |
|  | HCT116 | >> 16 | | 3.8 | 6.8 | 2.6 |

[a] Data are presented as the mean of independent experiments. "–" indicates not determined. Normoxia (21% O_2_); Hypoxia (induced by CoCl_2_). Light conditions: white LED, 220.7 mW/cm², 30 s.

[b] The IC50 value far exceeds the experimental endpoint concentration.

Figure S38 Representative fluorescence images of HCT116 cells after different treatments stained with Calcein-AM (green, live cells) and PI (red, dead cells). Scale bar: 200 μm. For light-treated groups, cells were irradiated with white light (220.7 mW/cm^2^) for 30 s.

Figure S39 Chemiluminescence imaging of model mice and normoxic mice at different time points after intraperitoneal injection of CL-Azo-CPT/Ce6 NPs (100 μM, 200 μL per mouse). Exposure time: 60 s.

Figure S40 Chemiluminescence imaging of model mice and normoxic mice at different time points after intraperitoneal injection of CL-Azo-CPT NPs (100 μM, 200 μL per mouse). Exposure time: 60 s.

Figure S41 The average chemiluminescent signal intensities from the same region of mouse over time (mean ± SD, *n* = 3).

Figure S42 Bioluminescence images of model mice (for CL-Azo-CPT/Ce6 NPs) after intraperitoneal injection of the luciferase substrate diphenylterazine (3 mM, 100 μL per mouse). Exposure time: 10 s.

Figure S43 Bioluminescence images of model mice (for CL-Azo-CPT NPs) after intraperitoneal injection of the luciferase substrate diphenylterazine (3 mM, 100 μL per mouse). Exposure time: 10 s.

Figure S44 Macroscopic photos of peri-gastric and mesenteric tumor nodules.

Figure S45 (A) Percentage of apoptotic cells calculated from the TUNEL results. (B) Percentage of Ki67 positive cells after treatment. Data are presented as mean ± SD (*n* = 3). ns: no significant, **P* < 0.05, ***P* < 0.01.

Figure S46 Serum biochemical analysis of kidney and liver function parameters. Data are expressed as mean ± SD (*n* = 3).

Figure S47 H&E staining reveals changes in major organs of mice after receiving different treatments. Key features are highlighted: epithelial cell necrosis (green arrows), lymphocytes (blue arrows), and congestion (yellow arrows). Scale bar: 100 μm.

**Reference**

[1]	L. Wei, Y. Wu, Y. Zhou, M. Yang, H. Li, W. C. Geng, Z. Yuan, J. Gao, *J Med Chem* **2025**, 68, 20506.

[2]	W. Fan, H. Peng, Z. Yu, L. Wang, H. He, Y. Ma, J. Qi, Y. Lu, W. Wu, *Acta Pharm Sin B* **2022**, 12, 2479.
